# DECIDE: a cluster randomized controlled trial to reduce non-medically indicated caesareans in Burkina Faso

**DOI:** 10.1186/s12884-016-1112-8

**Published:** 2016-10-21

**Authors:** Charles Kaboré, Valéry Ridde, Séni Kouanda, Ludovic Queuille, Paul-André Somé, Isabelle Agier, Alexandre Dumont

**Affiliations:** 1 Institut de recherche pour le développement (IRD), Université Paris Descartes, UMR 196 Centre Population et Développement (CEPED), Paris, France; 2 Institut de Recherche en Science de la Santé (IRSS), Ouagadougou, Burkina Faso; 3 Department of Preventive Medicine, University of Montreal School of Public Health (ESPUM) and University of Montreal Public Health Research Institute (IRSPUM), Montreal, Canada; 4 AGIR/ Groupe de travail en santé, Ouagadougou, Burkina Faso

**Keywords:** Non-medically indicated caesarean, Trial, Audit, Training, SMS-based reminders, SMS, Mixed methods, Process analysis, Burkina Faso

## Abstract

**Background:**

Since 2006, Burkina Faso has subsidized the cost of caesarean sections to increase their accessibility. Caesareans are performed by obstetricians, general practitioners, and nurses trained in emergency surgery. While the national caesarean rate is still too low (only 2 % in 2010), 12 to 24 % of caesareans performed in hospital are, in fact, not medically indicated. The objective of this study is to evaluate the effectiveness and analyze the implementation of a multi-faceted intervention to lower the rate of non-medically indicated caesareans in Burkina Faso.

**Methods:**

This study combines a multicentre cluster randomized controlled trial with an implementation analysis in a mixed-methods approach. The evidence-based intervention will consist of three strategies to improve the competencies of maternity teams: 1) clinical audits based on objective criteria; 2) training of personnel; and 3) decision-support reminders of indications for caesareans via text messages. The unit of randomization and of intervention is the public hospital equipped with a functional operating room. Using stratified randomization on hospital type and staff qualifications, 11 hospitals have been assigned to the intervention group and 11 to the control group. The intervention will cover 1 year. Every patient who delivered by caesarean during a 6-month period in the year preceding the intervention and the 6 months following its end will be included in the trial. The change in the rate of non-medically indicated caesareans is the main criterion by which the intervention’s impact will be assessed. To analyze the intervention process, a longitudinal qualitative study consisting of deliberative workshops and individual in-depth interviews will be conducted. The target outcome is a 50 % reduction in the rate of non-medically indicated caesareans.

**Discussion:**

This study will provide evidence regarding the effectiveness of a multi-faceted intervention for reducing non-medically indicated caesareans in a low-income country. By combining qualitative and quantitative methods, the study’s findings will allow understanding the factors that could influence the intervention process and ultimately the intended outcomes.

**Trial registration:**

The DECIDE trial is registered on the Current Controlled Trials website under the number ISRCTN48510263 on January 28, 2014.

**Electronic supplementary material:**

The online version of this article (doi:10.1186/s12884-016-1112-8) contains supplementary material, which is available to authorized users.

## Background

In Burkina Faso, although the national caesarean rate increased from 0.7 % in 2003 [1] to 2 % in 2010 [2], it remains well below the minimum rate of 3 % required to meet the needs [3]. The institutional caesarean rate has risen steadily since the introduction of a policy to subsidize emergency obstetric care in 2006, which significantly increased access to caesareans [4]. Under this policy, caesarean costs are subsidized up to 80 % (100 % in some districts) and transportation is fully funded between primary health centres and referral hospitals, where caesareans are performed. Similar policies have led to very high institutional caesarean rates in other sub-Saharan countries [5]. In fact, according to Burkina Faso’s Ministry of Health, the rate surpassed 40 % in some hospitals in 2013 [6]. Research has shown, however, that between 12 and 24 % of caesareans in that country are not medically indicated [7, 8].

In Burkina Faso, as in many African countries, excessive caesarean rates in some hospitals have been attributed to limited training and knowledge of personnel [7, 9]. In those facilities, it is not only obstetricians who assess indications for caesareans, but also general practitioners and nurses trained in emergency surgery (a 6-month training program involving 1 month of theoretical courses and a 5-month practicum in surgical and obstetric emergencies).

While greater access to caesareans is a necessity in countries where the national rate is below 10 % [10], it is imperative that quality improvement programs be implemented at the same time to prevent excessive and inappropriate increases in caesarean rates [11]. High rates of non-medically indicated caesareans (NMIC) are associated not only with adverse health outcomes for mothers and infants, but also with high public spending on health, presenting equity and efficiency challenges for low-resource countries [12–19].

Various strategies for reducing the proportion of NMICs have been tested and evaluated: 1) obtaining a second opinion on the indication for a caesarean before proceeding [20]; 2) encouraging normal, midwife-assisted deliveries outside the hospital [21, 22]; 3) establishing guidelines based on recommendations of professional associations [23, 24]; and 4) auditing indications for caesareans and providing feedback to health professionals, combined with instituting best practices for managing labour and performing caesareans [25]. A meta-analysis of 10 randomized controlled trials in high-income countries showed a 19 % reduction in caesarean rates using one or a combination of the different approaches cited above. The most effective strategies were clinical audits with feedback (RR = 0.87; 95 % CI = 0.81, 0.93), continuous quality improvement strategies (RR = 0.74; 95 % CI = 0.70, 0.77), and multi-faceted interventions combining several approaches (RR = 0.73; 95 % CI = 0.68, 0.79) [26, 27].

Furthermore, the use of SMS (Short Message Service) technology appears to have a positive, and less costly, impact on the continuing education of healthcare providers in sub-Saharan Africa [28–30]. Indeed, studies on its use have shown SMS is easy to implement and produces positive results in terms of improved knowledge and practices among health professionals in different contexts [31–33].

While the results of randomized controlled trials in high-income countries are encouraging [25], we found no evidence that these interventions, whether alone or in combination, are effective in reducing NMIC rates in low- or middle-income countries.

The objective of the DECIDE (Appropriate decision for caesarean section in Burkina Faso) trial is to evaluate the effectiveness and understand the implementation of an intervention combining three potentially effective approaches for reducing NMIC rates: 1) training in best practices during labour and delivery to favor vaginal delivery for low-risk women; 2) clinical audits based on objective criteria for the main indications for caesareans; and 3) SMS-based reminders to support decisions regarding clinically indicated caesareans.

### Hypotheses

Our main hypothesis is that clinical audits in healthcare facilities, combined with training and decision-support reminders to health professionals, will help reduce the NMIC rate by at least 50 %.

Our secondary hypotheses are that the intervention will improve: 1) health professionals’ knowledge; 2) the quality of caesareans; and 3) maternal and perinatal outcomes.

Regarding implementation, we posit that health workers will both appropriate and appreciate all three components of the intervention, and that any heterogeneity in results will be explained by the specific contexts of the different hospitals [34, 35].

## Methods

### Study design

This is a multicentre cluster randomized controlled trial of a complex intervention combined with an implementation analysis. To understand the reasons underlying the intervention’s effectiveness (or lack thereof), the heterogeneity of its effects, and role of context, it is essential that effectiveness and implementation (including fidelity) be analyzed together [36, 37]. This is a fundamental approach in operational research in the health field [38, 39]. To avoid contamination bias between clinicians in the same service, the unit of randomization and intervention is the hospital.

### Inclusion and exclusion criteria

A hospital was eligible for the trial if it had a functional operating room and had performed at least 200 caesareans in 2012. Twenty-two public hospitals were included in the trial between May and June 2014, out of a total of 24 eligible hospitals (91.6 %). Two university hospitals were excluded due to the high number of personnel in training. The 22 hospitals in the trial are representative of Burkina Faso’s current health system, spanning the full range in terms of context (urban versus rural), level of care (district and regional hospitals) and qualifications of health personnel. This process thus guaranteed a certain external validity of the results.

The intervention directly targets health professionals who provide obstetric care in the participating hospitals, while indirectly targeting the women giving birth there. The health professionals have been trained in the management of labour and delivery, with some also trained to perform caesareans. All health professionals involved in managing deliveries in their institutions were included in the study (*n* = 978). This total consisted of obstetricians and gynaecologists (*n* = 39), general practitioners (*n* = 32), physician anaesthetists (*n* = 3), nurses (*n* = 463), and midwives (*n* = 441). Maternity services personnel who were not involved in obstetric care in the labour room were excluded from the trial (*n* = 446).

The first 100 women with caesarean deliveries in the 22 hospitals during a 6-month period in the year preceding the intervention (May 2 to November 2, 2014) were included in the study. The first 100 women with caesarean deliveries in the 6 months following the end of the intervention will also be included. As the intervention will last 1 year, the pre- and post-intervention data collection periods will cover the same months of the year to avoid any seasonality bias. Women whose caesareans were performed in another hospital and who were subsequently transferred to a participating hospital will be excluded from the study.

### Activities in the intervention group

**Fig. 1:**
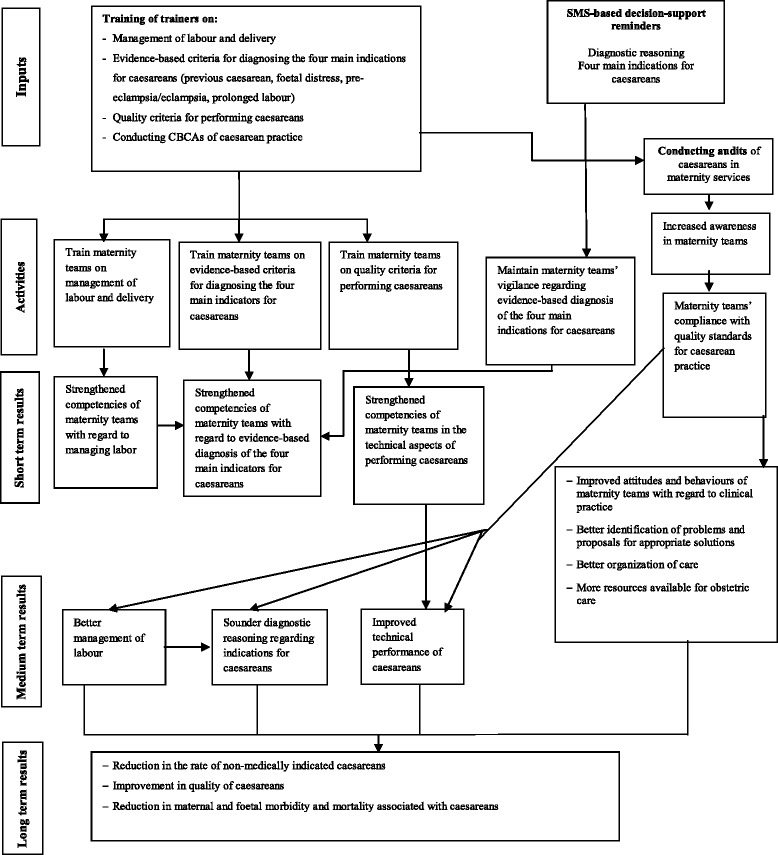
show the logic of the intervention

The activities will be conducted from May 2015 to April 2016. The sequence of activities over the 12 months will be directly focused on developing local leadership and strengthening the obstetric teams’ capacities. To achieve this goal, the intervention will be implemented in several stages. Figure 1 shows the logic of the intervention [40–42].

The intervention will begin with the training of local trainers on: 1) evidence-based standards for the management of labour, the reasoning used to diagnose the main indications for caesareans, and the quality of the surgical procedure; and 2) conducting clinical audits of indications for caesareans based on objective criteria (criteria-based clinical audits, CBCA). The trainers will create CBCA teams in their own hospitals and will organize training for obstetric teams on best practices. With a view to sustainability, no financial incentive will be given to local trainers or to health professionals who undergo the training in the intervention hospitals. Thereafter, decision-support reminders of evidence-based criteria for diagnosing the main indications for caesareans, conveyed via SMS, will be used to supplement the staff training.

The intervention consists of five stages:

1. *Training the trainers:* The local trainers are the chiefs of maternity services in the intervention hospitals (one physician per hospital). In May 2015, the trainers attended a 3-day training session led by two experts from the Society of Gynaecologists and Obstetricians of Burkina (SOGOB). The training was based on the WHO guidelines for managing complications of pregnancy and childbirth [43] and clinical decision algorithms that had been developed as part of this study (see Additional files 1, 2, 3, 4, 5 and 6). The session included 2 days of training on: 1) the diagnostic reasoning involved in identifying the main indications for caesareans (previous caesarean, prolonged/obstructed labour, pre-eclampsia/eclampsia, foetal distress); 2) the quality of the surgical procedure; and 3) the decision algorithms validated by the experts. A third day was allocated to training on conducting CBCAs.
2. *Establishing and training a multidisciplinary audit committee in each intervention hospital:* Setting up these CBCA committees (consisting of physicians, midwives, nurses, and administrators) will involve: a) identifying and training people who will be responsible for collecting data on caesareans; and b) training committee members on how to conduct CBCAs.
3. *Setting up the audit cycle:* In each intervention hospital, the audit committee will implement CBCAs according to the approach proposed by WHO [44]. Monthly audit meetings are recommended to analyze the caesareans performed in the health facility. A caesarean audit guide prepared as part of this study (see Additional file 7) will be distributed to each audit committee member. It describes the steps involved in conducting an audit, from preparation all the way to closing the session, offers proposals regarding the roles of the different actors involved in the process, and suggests various media and data sheets needed to conduct audits.
4. *Training of maternity teams on best practices:* The local trainers will train the maternity teams in each hospital. First they will assess the needs for training in best practices. Then they will organize four training sessions on best practices (one session for each of the four main indications) during the intervention period. The trainers will select the topics based on areas in need of improvement, as identified in the audit meetings. As such, the content will be tailored to local contexts and situations.
5. *Decision-support reminders:* Health professionals will receive weekly decision-support reminders regarding diagnostic reasoning and the relevance of indications for caesareans. This strategy will be applied from the moment best practices training begins and audits are conducted, all the way to the end of the intervention period. The reminders will be sent on workdays, in the afternoons, via SMS messages. Once a week, each health professional will receive a clinical recommendation (see Additional file 8) on the diagnostic reasoning regarding the selected caesarean indications (previous caesarean, prolonged/obstructed labour, pre-eclampsia, eclampsia, foetal distress). Diagnostic reasoning criteria were established from the reference documents that were used for training and validated by expert consensus.

### Control group

No intervention is planned for the control group as part of this project.

### Implementation analysis

The intervention has been carefully planned and is evidence-based, but to be effective it must be correctly implemented [45]. Thus, we will need not only to verify implementation fidelity but also to understand its process. To do this, we will use a two-pronged strategy.

The first component, *implementation monitoring,* will involve monitoring and facilitating implementation of the intervention’s different steps. Monitoring will be done via quarterly visits to each intervention hospital in which the following criteria will be assessed: a) training schedule respected and attendance list available; b) decision algorithms available; c) schedule of audit meetings respected and attendance list available; d) audit recommendations implemented; and e) decision-support SMS message system functional.

We will inventory all training provided to staff in the maternity services of the hospitals being monitored (topics, content, number of participants and their profiles, duration, organizer). This information will be recorded on a training census form in collaboration with the chiefs of maternity services. We will also document any movement of personnel from the intervention group to the control group.

The *qualitative component* will consist of four stages (see additional file 9):

1. Analysis of the pre-intervention situation
  This analysis is focused on NMIC rates and their inter-hospital variability. In the train-the-trainers workshop, information on overall NMIC rates and their variability among hospitals (anonymized) was presented to solicit the perceptions of the group as a whole (*n* = 1, 11 participants) and then of each participant in individual interviews (*n* = 11).
2. Analysis of the implementation of the intervention
  For this, 3 months after the train-the-trainers workshop at the start of the intervention, the analysis of the overall findings of the study on caesareans and on variations in NMIC rates will be repeated using data collected from groups and individuals (*n* = 22), i.e., two persons per hospital who decide on caesareans and did not attend the first workshop. Then, in months 6 and 12 of the intervention and in each hospital (*n* = 11), we will conduct group discussions, as well as non-participant observation of an audit committee meeting and interviews with two health professionals who decide on caesareans (*n* = 22). The health professionals’ views on the relevance and effectiveness of the intervention strategies will be collected, as well as their opinions regarding the various factors that could influence, either positively or negatively, the intervention process and ultimately the intended outcomes.
3. Analysis of events in the control group
  For this analysis, group discussions with the maternity teams of each control hospital are planned for the 12th month of the intervention. These discussions will focus on NMIC rates and their variability among the hospitals. A summary will also be prepared of any significant events that have occurred in the maternity service over the intervention period, especially any training provided to health professionals. The aim is to take into account any event that might influence the key indicator of the intervention’s impact.
4. Analysis of the potential for transferring the intervention outcomes to other contexts
  Individual interviews will be conducted with key stakeholders (health professionals, trainers, and the principal investigator) using an analytical framework developed specifically for this purpose [46].

### Assessment criteria

These criteria were measured for a 6-month period (May 2–November 2, 2014) preceding the intervention year and will be measured again in the 6 months following the end of the intervention, corresponding to the same months of the year.

The primary assessment criterion is the NMIC rate.

We conducted a literature review to discern the diagnostic reasoning underlying evidence-based indications for caesareans. The four main indications for caesareans in Burkina Faso are: previous caesarean, prolonged/obstructed labour, pre-eclampsia, and acute foetal distress. In the 22 participating hospitals in the pre-intervention study, these four indications accounted for 76 % of all caesareans performed. The relevant references [47–70] were consulted and used to generate a provisional list of good practice criteria. Preference was given to evidence obtained through randomized controlled trials, considered the most rigorous. The provisional list of criteria was sent to 16 international and national experts (gynecologist-obstetricians, midwives, and a public health physician, see Additional file 10). The experts gave their opinions on the relevance of each criterion and proposed others. The criteria retained were those validated by at least two-thirds of the experts. We then developed clinical decision trees (algorithms) for managing the four main indications for caesareans and derived the criteria for NMIC.

The NMIC criteria are presented in Table 1. For each indication, all the criteria are considered simultaneously. A caesarean is judged to be non-medically indicated when all the criteria are met or when they are not documented and no opposing medical indication is provided. The NMIC rate was calculated for each hospital. This rate is the proportion of NMICs among all the caesareans performed for the four indications during the same period.

**Table 1:** Criteria for non-medically indicated caesareans ^a^

Previous caesarean section	Prolonged/obstructed labour
Pre-labour caesarean not indicated if ^a^: - One previous caesarean section with transversal scar - Singleton foetus in cephalic presentation - Lack of ultrasound or clinical evidence of macrosomia - Lack of radiographic or clinical evidence of restricted pelvis - Possibility of performing emergency intra-partum caesarean section 24 h on call Caesarean during labour not indicated if ^a^: - Same criteria as pre-labour caesarean (see above) - Cervical dilation progressing normally (>2 cm in 4 h) - Presentation progressing normally at full dilation (no arrest >3 h) - Lack of evidence of foetal distress^b^ - No documented signs of uterine rupture OR Duration between full dilation and decision for caesarean delivery >3 h and no attempt of instrumental delivery (forceps or vacuum) if this is indicated	Pre-labour caesarean for presumed obstructed labour not indicated if ^a^: - Singleton foetus in cephalic presentation - Lack of ultrasound or clinical evidence for macrosomia - Lack of radiographic or clinical evidence of restricted pelvis - No documented history of fistula or uterine malformation - No documented history of uterine rupture Caesarean for failed induction or slow dilation without foetal distress is not indicated if ^a^: - Membranes intact OR - Membranes ruptured, uterine contractions adequate and time elapsed since stagnation of dilation under 4 h OR - Membranes ruptured, uterine contractions inadequate, no attempt at augmentation with oxytocin OR - Membranes ruptured, uterine contractions inadequate, augmentation with oxytocin and time elapsed since stagnation of dilation under 6 h Caesarean at full cervical dilation without foetal distress not indicated if ^a^: - Non-engagement of presentation and time elapsed between full dilation and decision for caesarean under 3 h - Engaged head and instrumental delivery (forceps or vacuum) not attemped
Pre-eclampsia	Foetal distress
Pre-labour caesarean not indicated if ^a^: - Lack of evidence of foetal distress^b^ - Lack of clinical or ultrasound evidence of foetal growth restriction - No documented signs of severity for the woman^c^ - No documented signs of abruptio placentae Caesarean during labour not indicated if ^a^: - Same criteria as pre-labour caesarean (see above) - Cervical dilation progressing normally (>2 cm in 4 h) - Presentation progressing normally at full dilation (no arrest >3 h)	Caesarean during labour not indicated if ^a^:signs of abruptio placentae - Clear amniotic fluid or not documented -No maternal fever or not documented - Cervix dilation or presentation progressing normally - Foetal heart rate normal (120–160 beats/min.) OR abnormal but uncorrected (oxygen administration and mother on left side)

^a^All criteria are required

^b^Signs of foetal distress: foetal heart rate abnormalities (<120 or >160 beats/min. or repeated decelerations); coloured amniotic fluid

^c^Signs of severe pre-eclampsia: blood pressure ≥160/110 mmHg; albuminuria ≥3+ or ≥3 g/24 h; oliguria <30 mL/h; headache; epigastric pain; vision disorders; neurologic disorders; seizures; hemolysis; low platelet count; high liver enzymes

Secondary assessment criteria are:

- *Ratio of pre-/post-labour caesareans*
  Caesareans fall into three groups: 1) those planned and performed before labour; 2) urgent caesareans performed before labour; and 3) urgent caesareans performed during labour. It is expected that improvements in intrapartum care quality would be accompanied by a decrease in urgent caesareans during labour and an increase in pre-labour caesareans—hence, a reduction in the ratio of pre-/post-labour caesareans [71].
- *Health professionals’ knowledge score*
  We developed short case histories (vignettes) to measure health professionals’ knowledge in terms of their ability to correctly apply the main indications for caesareans: previous caesarean, pre-eclampsia, prolonged/obstructed labour, foetal distress. These knowledge/skills criteria were based on the diagnostic reasoning criteria for indications as selected by the experts. Each professional’s knowledge score corresponds to the number of correct responses for the 33 vignettes.
- *Caesarean quality score*
  In addition to the diagnostic reasoning criteria for caesarean indications, the expert committee also developed good-practice criteria to assess whether the caesarean was performed at the proper time and appropriately. Those criteria are presented in Table 2.

**Table 2:** Caesarean quality criteria

ᅟ	ᅟ
− Patient must be given clear information on the indication for the caesarean. − Elapsed time between decision and incision must be no longer than 30 min. − Preference is given to using local or regional anaesthesia. − Patient receives prophylactic antibiotics. − Misgav Ladach surgical technique is used. − Joel-Cohen skin incision is used. − Delivery is done using controlled cord traction rather than manually. − In post-operative care, patients without complications resume liquid diet after 6 h. − In post-operative care, high-risk patients (morbid obesity, pre-eclampsia, history of thromboembolism) undergo thromboembolism prevention therapy.
  In each hospital, all caesareans performed over the same given period will be evaluated. One point will be attributed for each criterion met (0 for those not met). For each case, total points attributed will be divided by the total number of criteria, giving a score of between 0 and 100 %. Each hospital’s quality score will be the average of the scores for all the caesareans evaluated for that hospital.
- *Resource availability score*
  Resource availability indicators will be measured in each hospital before and after the intervention through a systematic and standardized inventory of available resources. For each facility, a hospital environment complexity index will be calculated to reflect the availability of the different categories of resources needed to provide good-quality obstetric care [72, 73]: basic services; screening tests; basic emergency obstetric resources; intrapartum care; general medicine services; anaesthesiology; human resources; teaching and clinical resources; and clinical care protocols.
- *Perinatal mortality in infants delivered by caesarean*
  We selected this indicator because we believe improvements to obstetric care prompted by audits should also have an impact on perinatal outcomes related to intrapartum care [65, 73]. Included in the definition are immediate neonatal deaths (within 24 h after birth) and fresh stillbirths; excluded are macerated stillbirths, deaths of premature infants (less than 37 weeks amenorrhea), and deaths of severely deformed infants. Perinatal mortality related to intrapartum care is defined as the number of immediate deaths and fresh stillbirths divided by the number of non-preterm caesarean deliveries in the same period.
- *Intra-hospital maternal mortality among patients with caesareans*
  This indicator was selected because we believe that, as in perinatal deaths, improvements in caesarean practice prompted by audits should also have a positive impact on maternal outcomes [73–75]. This indicator is defined as the ratio between the number of post-caesarean maternal deaths occurring in a hospital during the study period and the number of caesareans performed in that hospital over that period.

### Randomization and allocation

In February 2015, after a 6-month pre-intervention study, hospitals were allocated randomly to either the intervention or control group. Participating hospitals were grouped into three strata corresponding to different types of hospitals: regional hospitals, district hospitals in the two largest cities (Ouagadougou and Bobo Dioulasso), and district hospitals outside those two cities. All participating hospitals were randomly allocated simultaneously to minimize the risk of allocation bias [76, 77]. To prevent imbalance regarding the level of care in each stratum, block randomization was used to generate the allocation sequence [76, 77]. Investigators were informed of the allocation just before the intervention was implemented in the intervention group.

### Data collection and processing

A data collection system has been set up in each of the 22 participating hospitals. This system is inspired by the WHO global survey on maternal and perinatal health [73], which considers both individual clinical data and organizational data at the health facility level. Information on the first 100 caesareans performed in the 6-month pre-intervention period and the first 100 performed in the 6-month post-intervention period in each hospital will be entered by local data collectors (midwives trained for this task). These midwives fill out a standard form for each eligible caesarean, which includes information on maternal characteristics, diagnosed complications, prenatal care, management of labour, caesarean indication and procedure, post-operative monitoring, and maternal and infant vital signs at discharge (see caesarean form in Additional file 11). This information will be obtained from hospital registers and from available medical records, whose quality and archiving are regularly monitored by the study coordinator. Data will be collected daily during both the pre- and post-intervention periods. With regard to organizational data and the knowledge evaluation questionnaire, the study coordinator will compile an inventory of the resources available in all the hospitals and will administer the questionnaires to health professionals before and after the intervention period.

### Data management

Midwives trained for this task will collect information on caesareans daily using a form designed for this purpose, then will enter it into a mobile telephone using Pendragon Forms VI software. The electronic forms will then be transmitted via the telephone network to a server at the trial coordination centre at the University of Montreal, where they will be stored in a secure location.

### Data quality control

The quality of clinical data will be controlled in two steps. The first consists of quarterly visits by the study coordinator, who will verify that the data collection is exhaustive by comparing the number of eligible patients from the delivery register and the hospital’s surgical protocols register with the number of patient forms completed. An additional procedure will be carried out to check that the data entered in the forms matches the data in the source documents (medical records, delivery registers, surgical registers). The coordinator will verify the quality of the data collected in each form. The completeness rate will be estimated as the proportion of forms that contain 100 % of the following information: entry date, patient identity, method of admission, reason for admission, history of pregnancy, patient’s medical history, details of the obstetric examination and labour management, indication for the caesarean, technique used for the caesarean, post-operative monitoring, date of discharge, maternal and infant vital signs at discharge. The matching rate will be estimated as the proportion of forms containing information that is consistent with the source documents. When information is missing or inaccurate on any forms, the coordinator will correct those forms from the source documents in the presence of the midwife. The corrections will be made first on the paper form and then on the electronic form. Once the corrections have been made, the database will be updated. A second check for missing or abnormal data will be performed at the coordination centre and the data collected will be updated again. If necessary, missing or inaccurate information will be added and/or corrected by communicating with the midwives by telephone. To ensure data quality, there will be regular contact with the midwives (equipped with mobile telephones), who will be given lists of duplicates and missing or abnormal data and will be responsible for correcting these errors.

### Sample size calculation

This is a superiority trial, in which the intervention will be considered effective if it leads to a reduction of at least 50 % in the NMIC rate. The sample size was calculated to maximize statistical power [78]. With an α risk of 5 % (two-sided testing) and 1-β power of 90 %, and a variance inflation factor (VIF) = 3 (calculated using an average of 100 caesareans per hospital and an intra-cluster correlation coefficient (ρ) estimated at 0.02), 2,200 caesarean records and 22 hospitals are needed.

### Analysis strategy

For evaluating the effectiveness of clinical audits in reducing NMIC rates, the unit of analysis is the patient. The main tests will be intention-to-treat analyses. A first descriptive analysis will be done to verify group comparability in terms of the characteristics of hospitals and patients included in the pre-intervention period. The primary analyses will take into account inter- and intra-cluster variability. We will use generalized estimating equations (GEE) to adjust the variance estimation for the intervention’s effect on inter-dependence of the measured events (NMIC or not) among patients of the same hospital [79]. The exchangeable structure of the residual covariance matrix will be used to represent the intra-cluster correlations. The same approach will be used to test the effectiveness of CBCAs in relation to resource improvement (complexity score), medical practices (caesarean quality score), and reductions in maternal and perinatal mortality. The intervention’s effect on the NMIC rate will be estimated from the odds ratio (95 % CI) based on the GEE approach and tested with the two-sided Wald test (α = 0.05) [79]. The intervention’s effects will be evaluated systematically both without adjustment and with adjustments on the stratification variables (type of hospital) and on the other baseline characteristics selected a priori, whose distribution could, even with randomization, be unbalanced between the two groups.

Preliminary analyses will test whether the intervention’s effects vary according to hospital type, using chi-squared likelihood ratio tests with 2 degrees of freedom for hospital–intervention interaction. If the test does not reject the null hypothesis with α = 0.05, the interactions will be eliminated from the final model, which will enable the overall effect of the intervention to be estimated. However, if the interactions are statistically significant, subgroup analyses will be done to estimate the effects of the intervention separately for each type of hospital (district vs. regional), with a power of less than 90 %. A similar approach will be used to test whether the intervention’s effects depend on: 1) the caesarean rate in the pre-intervention period; 2) the initial knowledge score in the pre-intervention period; or 3) patient characteristics (age, parity, previous caesarean, prenatal care, referral for delivery) and any other variables distributed unevenly among groups in the pre-intervention period. In all secondary analyses, the intervention’s effects will be estimated from the adjusted odds ratio using the GEE approach, which generalizes the logistic regression to clustered data [79]. To analyze the implementation and heterogeneity of effects, interviews will be fully transcribed into a word processor program. Subsequently, qualitative data will be coded and organized using QDA Miner software and then subjected to content analysis [80].

### Type of analysis and treatment of hospitals lost to follow-up

We believe the acceptability of the various components of the intervention, including the caesarean audits, is high [34, 35]. Nevertheless, some hospitals may decide to withdraw from the study. For these hospitals, data collection will continue until the end of the study, in accordance with the commitment made by the hospital authorities at the time of inclusion. These hospitals will therefore not be excluded from the analysis. In accordance with the intention-to-treat analysis approach, every patient will be analyzed at the hospital where she was admitted, and each hospital will be analyzed in the group to which it was randomly allocated.

There is no data monitoring committee and no stopping rules.

### Potential limitations of the trial

Although there is no intervention planned as part of this study in the control group, activity related to data collection may have an impact on improving the practice of caesareans. However, this data collection effect is, a priori, similar in both groups (intervention and control). The results of our trial could be contaminated if there is a transfer of staff from intervention hospitals to control hospitals. Such contamination could reduce the apparent effectiveness of the intervention. To assess the extent of this potential contamination, we will keep track of all personnel transfers between hospitals of the two arms of the trial. It should be noted, however, that implementing the components of the intervention requires sustained teamwork and is unlikely to be accomplished by isolated individuals. We also plan to study in depth the events and environments of all hospitals in the control group. As the subsidy for emergency obstetric and neonatal care was passed until 2015, a decision not to extend it into 2016 could also limit this study.

**Start of study:** May 2, 2014

**Intervention period:** May 2015 to April 2016.

**End date:** November 2016

## Discussion

While greater access to caesareans is a necessity in countries where the national rate is below 10 % [10], it is imperative that quality improvement programs are implemented at the same time to prevent excessive and inappropriate increases in caesarean rates [11]. High rates of non-medically indicated caesareans (NMIC) are associated not only with adverse health outcomes for mothers and infants, but also with high public spending on health, presenting equity and efficiency challenges for low-resource countries [12–19].

Various strategies for reducing the proportion of NMICs have been tested and evaluated, while the results of some observational studies carried out in sub-Saharan Africa are promising [27], and the results of randomized controlled trials in high-income countries are encouraging [25], we found no evidence that these strategies are effective in reducing NMIC rates in low- or middle-income countries, nor on their large-scale implementation. For this reason, we think that this trial is relevant in the context of Burkina Faso where fees for caesarean delivery were partially removed in 2006, and then totally removed in 2016.

This study will provide evidence regarding the effectiveness of a multi-faceted intervention for reducing non-medically indicated caesareans in a low-income country. Although there is no “intervention”, the activity related to data collection may have an impact on labour and delivery management and on the indication of caesarean deliveries in the control group. However, this “data collection” effect is a priori similar in the two groups (intervention versus control). Furthermore, the implementation of audit activities may vary between hospitals and then the effect of the intervention may be different in various settings. By combining qualitative and quantitative methods, the study’s findings will allow understanding the factors that could influence the intervention process and ultimately the intended outcomes. The results of this study will be shared with health professionals and decision-makers in Burkina Faso and may also interest others low-income countries where the policy of user fees removal has been implemented and where indications for caesareans are not only assessed by obstetricians.

Participating hospitals were included on the basis of informed consent given by local authorities (hospital director and maternity service chief). The authorities were informed that: 1) all hospitals are free to withdraw from the project at any time; 2) the intervention will be offered in hospitals in the control group at the end of the study if proven effective; and 3) data collection will continue until the end of the study, even for hospitals that withdraw from the study. The collection of clinical data from hospital registers and medical records has been authorized by the hospital administrations and does not require patient consent [73].

All information collected on patients, health professionals, and health facilities is confidential. Access to the clinical database is restricted to the data manager until the end of the study. Access to the other databases is restricted to the researchers in charge of the study. For this study, interviews and observations will be conducted, and informed consent will be sought from all participants by means of information sheets, which they will sign. Given the variety of research activities, various types of information sheets will be developed. Consent will be certified in writing.

## Additional files

Additional file 1: Algorithm vaginal birth after caesarean section. (PDF 138 kb)
Additional file 2: Algorithm Eclampsia. (PDF 132 kb)
Additional file 3: Algorithm Pre-eclampsia. (PDF 192 kb)
Additional file 4: Algorithm Prolonged labor. (PDF 90 kb)
Additional file 5: Algorithm Fetal distress. (PDF 90 kb)
Additional file 6: Algorithm Contra-indication to vaginal birth. (PDF 166 kb)
Additional file 7: Caesarean audit guide. (DOC 564 kb)
Additional file 8: SMS-based reminders. (DOC 33 kb)
Additional file 9: Qualitative study tools. (DOC 51 kb)
Additional file 10: List of experts. (DOC 26 kb)
Additional file 11: Caesarean section form. (DOC 285 kb)

## Abbreviations

CBCA: Criteria based clinical audit
CEPED: Centre Population et Développement
CS: Caesarean section
ESPUM: University of Montreal School of Public Health
IRD: Institut de Recherche pour le Développement
IRSPUM: University of Montreal Public Health Research Institute, Montreal, Canada
IRSS: Institut de Recherche en Sciences de la Santé
NMIC: Non-medically indicated caesarean section
