# Supplementary figures and images for: DECIDE: a cluster randomized controlled trial to reduce non-medically indicated caesareans in Burkina Faso
Source: BMC Pregnancy Childbirth. 2016 Oct 21;16:322. doi: 10.1186/s12884-016-1112-8 (PMC5073955; doi:10.1186/s12884-016-1112-8)

## Algorithm: Vaginal birth after caesarean (VBAC)

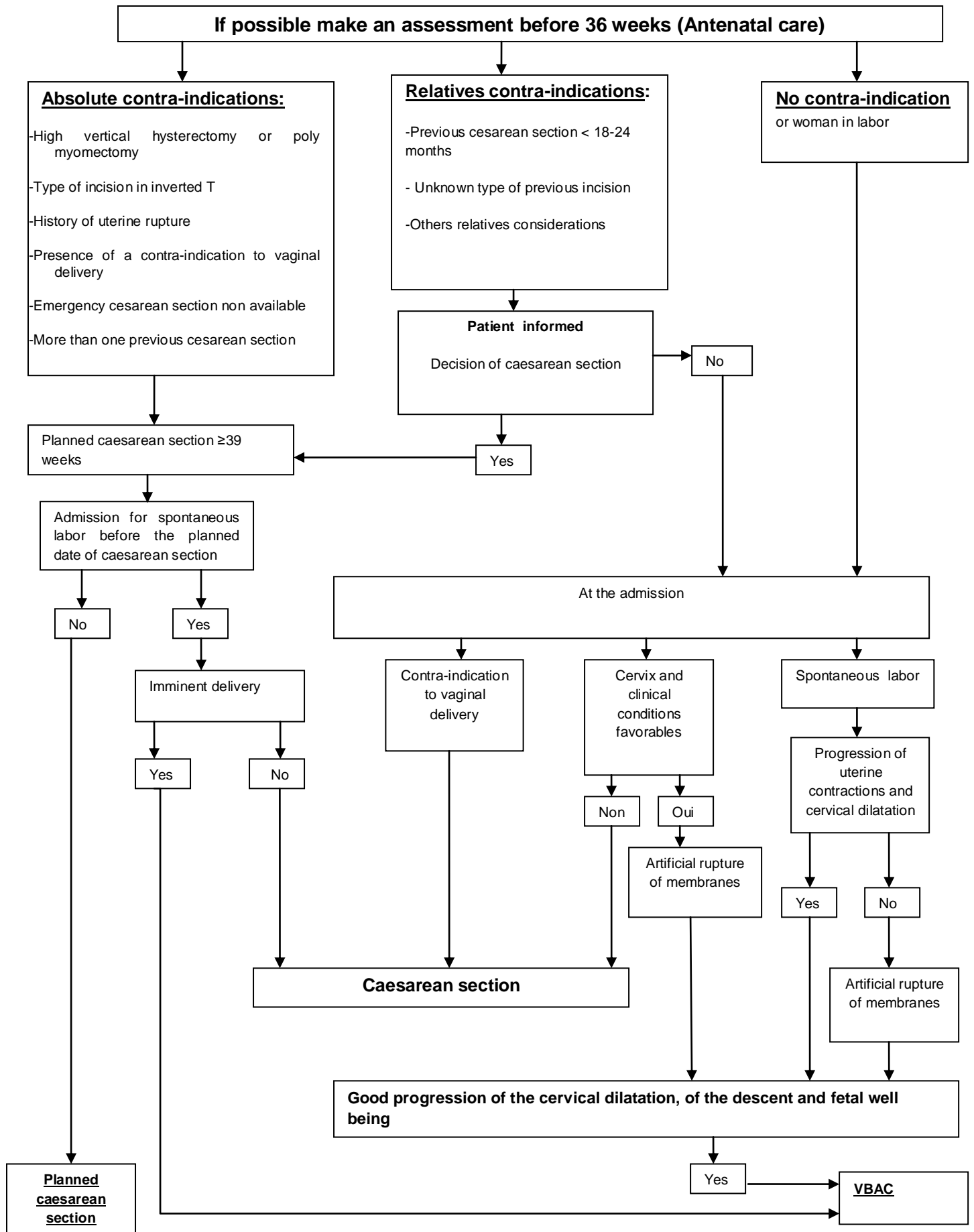

Supplement: Additional file 1: — Algorithm vaginal birth after caesarean section. (PDF 138 kb) [file 12884_2016_1112_MOESM1_ESM.pdf]

## Algorithm : Prolonged labor

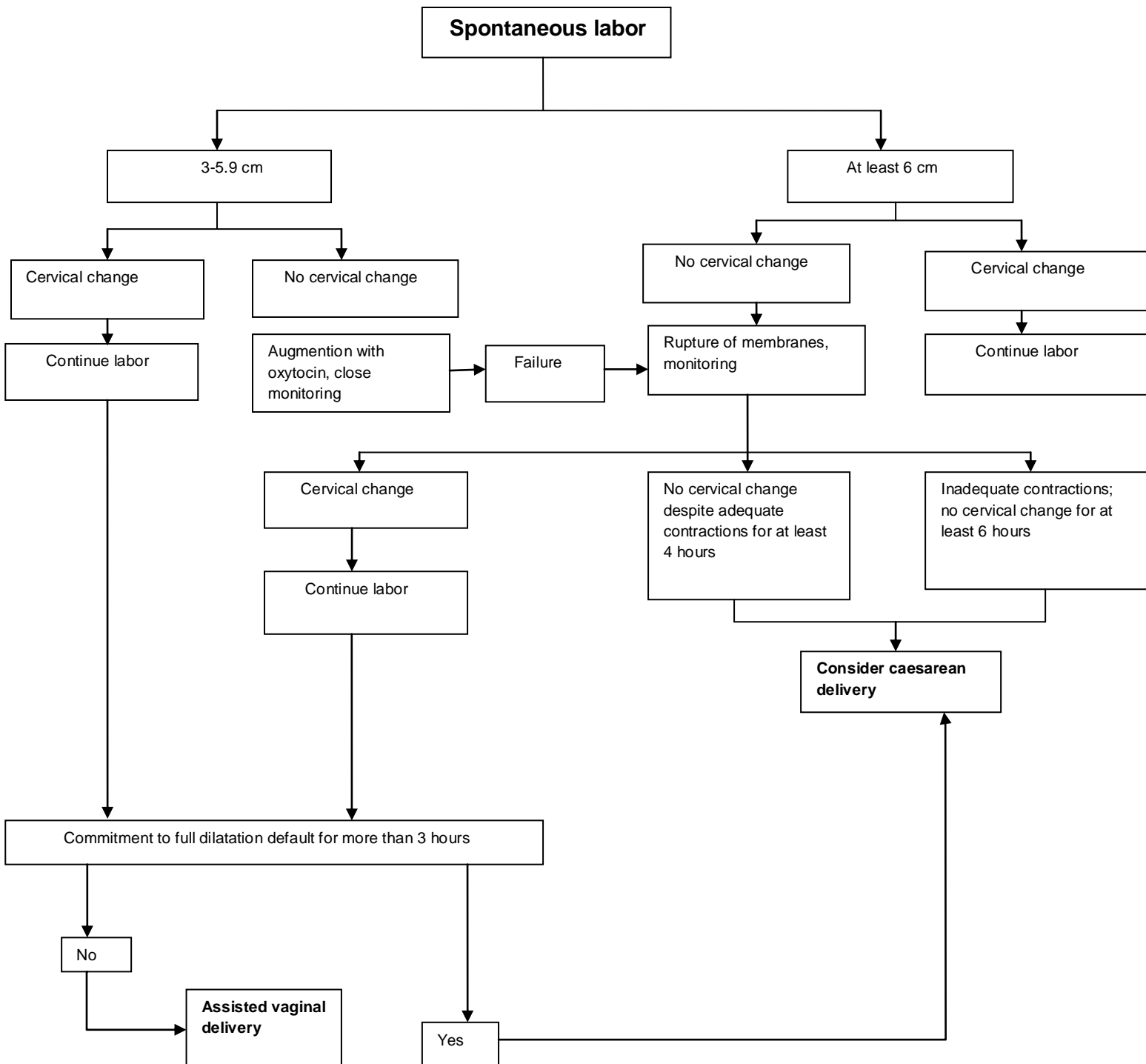

Supplement: Additional file 4: — Algorithm Prolonged labor. (PDF 90 kb) [file 12884_2016_1112_MOESM4_ESM.pdf]

## Algorithm: Fetal distress during labor

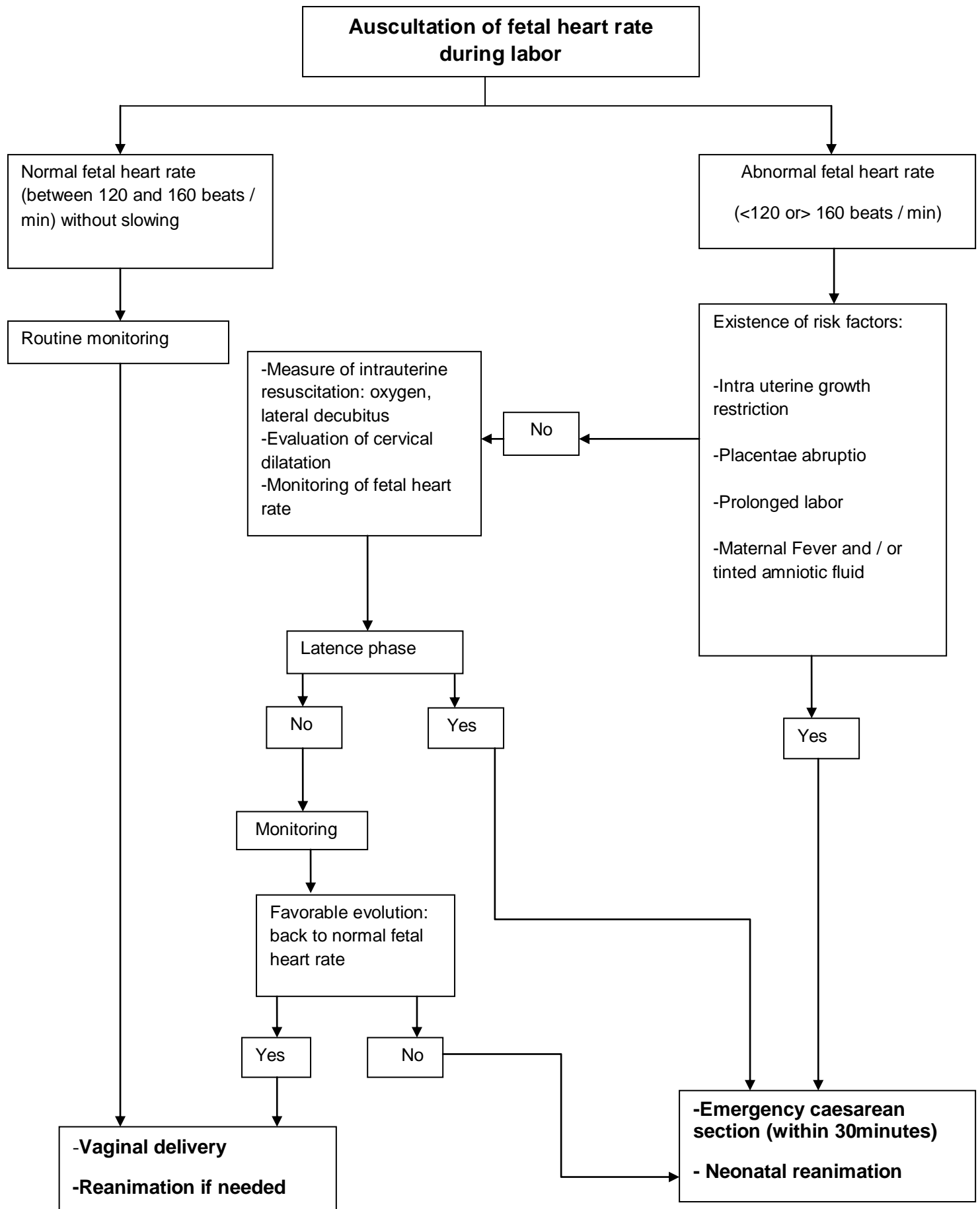

Supplement: Additional file 5: — Algorithm Fetal distress. (PDF 90 kb) [file 12884_2016_1112_MOESM5_ESM.pdf]
