# Supplementary material for: DECIDE: a cluster randomized controlled trial to reduce non-medically indicated caesareans in Burkina Faso
Source: BMC Pregnancy Childbirth. 2016 Oct 21;16:322. doi: 10.1186/s12884-016-1112-8 (PMC5073955; doi:10.1186/s12884-016-1112-8)
Supplement: Additional file 2: — Algorithm Eclampsia. (PDF 132 kb) [file 12884_2016_1112_MOESM2_ESM.pdf]

Algorithm : ECLAMPSIA

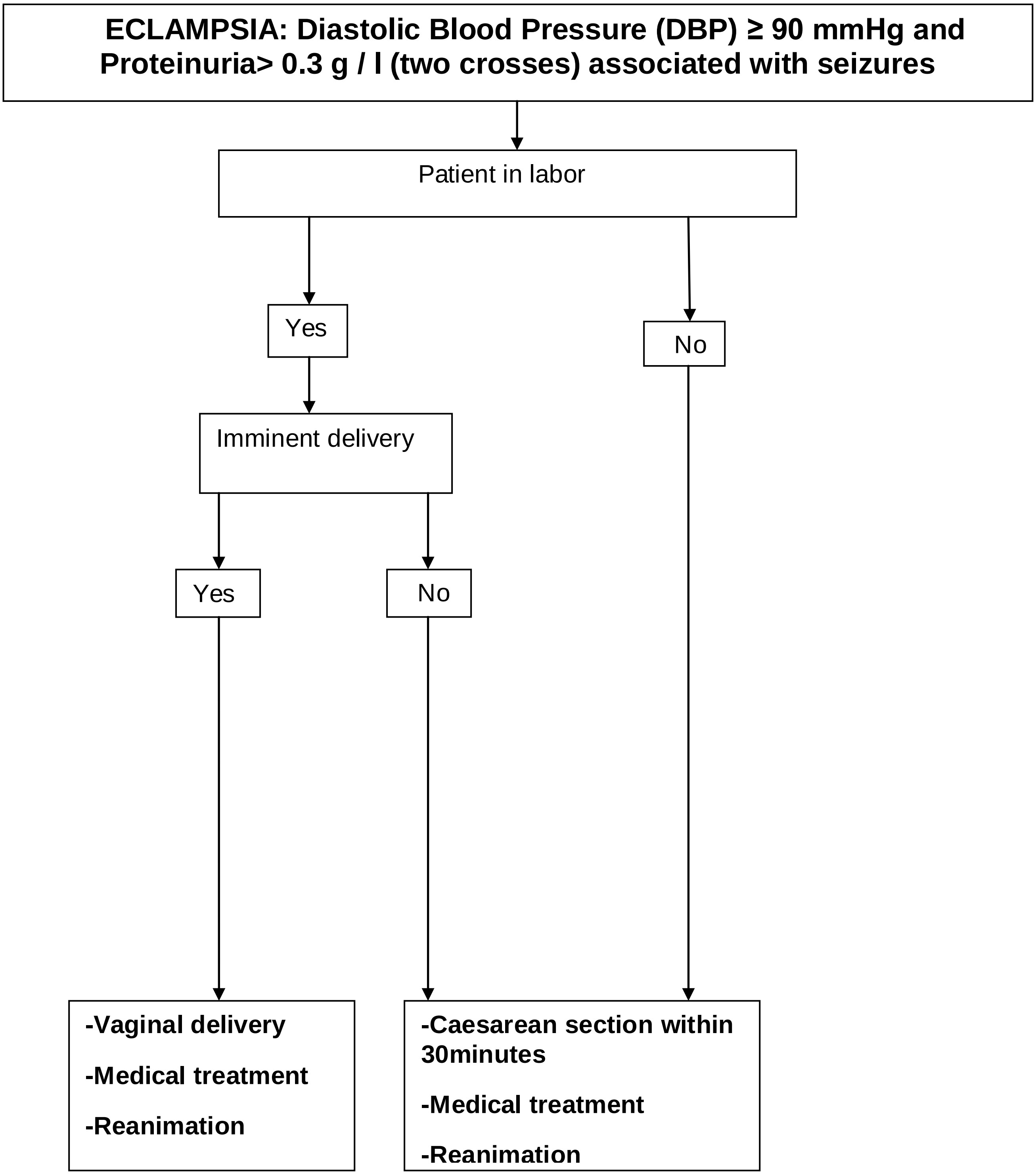

Source: Recommendations for clinical practice of the African Society of Gyneacology and Obstetrics (SAGO) and opinions of the group of experts
