# Supplementary material for: DECIDE: a cluster randomized controlled trial to reduce non-medically indicated caesareans in Burkina Faso
Source: BMC Pregnancy Childbirth. 2016 Oct 21;16:322. doi: 10.1186/s12884-016-1112-8 (PMC5073955; doi:10.1186/s12884-016-1112-8)
Supplement: Additional file 7: — Caesarean audit guide. (DOC 564 kb) [file 12884_2016_1112_MOESM7_ESM.doc]

| 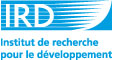 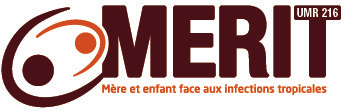 |
| --- |
| Manual for caesarean section audit |
| DECIDE trial |
|  |

Authors: Charles Kabore*, Alexandre Dumont*

* UMR 216 MERIT, Institut de recherche pour le développement

Université René Descartes, 4 avenue de l’Observatoire, 75006 Paris

|  |
| --- |

**Summary**

[Context 2](#__RefHeading___Toc438738822)

[Caesarean section in sub-Saharan Africa 2](#__RefHeading___Toc438738823)

[Interventions to reduce caesarean section rates 3](#__RefHeading___Toc438738824)

[OBJECTIVES OF THE GUIDE 5](#__RefHeading___Toc438738825)

[CLINICAL AUDITS IN GENERAL 6](#__RefHeading___Toc438738826)

[1. DEFINITIONS 6](#__RefHeading___Toc438738827)

[2. THE AUDIT CYCLE 6](#__RefHeading___Toc438738828)

[Step 1: Establishment of good practice criteria 6](#__RefHeading___Toc438738829)

[Step 2: Measure of the current practice 7](#__RefHeading___Toc438738830)

[Step 3: Objectives to achieve 7](#__RefHeading___Toc438738831)

[Step 4: Changes to operate 8](#__RefHeading___Toc438738832)

[3. PRINCIPLES OF AUDITS 8](#__RefHeading___Toc438738833)

[4. INFLUENCE FACTORS OF AUDITS 9](#__RefHeading___Toc438738834)

[4.1. The facilitating factors 9](#__RefHeading___Toc438738835)

[4.2. Obstacles 9](#__RefHeading___Toc438738836)

[4.3. The risks 9](#__RefHeading___Toc438738837)

[CRITERIA BASED CLINICAL AUDIT (CBCA) 10](#__RefHeading___Toc438738838)

[1. DEFINITIONS 10](#__RefHeading___Toc438738839)

[2. ESTABLISHMENT OF AN AUDIT TEAM 10](#__RefHeading___Toc438738840)

[3. SELECTION OF THEMES, OPERATIONAL DEFINITIONS AND GOOD PRACTICE CRITERIA 12](#__RefHeading___Toc438738841)

[a) Select a topic audit 12](#__RefHeading___Toc438738842)

[b) Establish operational definitions 12](#__RefHeading___Toc438738843)

[c) Identify good practice criteria 13](#__RefHeading___Toc438738844)

[d) Identify sources of information 14](#__RefHeading___Toc438738845)

[e) Develop structured audit forms 14](#__RefHeading___Toc438738846)

[4. ANALYSIS OF THE CURRENT PRACTICE 15](#__RefHeading___Toc438738847)

[a) The number of cases 15](#__RefHeading___Toc438738848)

[b) Identification and retrieval of medical records 15](#__RefHeading___Toc438738849)

[c) Data extraction from medical records 16](#__RefHeading___Toc438738850)

[d) Data Analysis 16](#__RefHeading___Toc438738851)

[5. CONCLUSIONS AND OPPORTUNITY TO SET OBJECTIVES 18](#__RefHeading___Toc438738852)

[Develop an action plan 18](#__RefHeading___Toc438738853)

[6. IMPLEMENTATION OF THE PLAN OF ACTION 19](#__RefHeading___Toc438738854)

[a) Assign roles and responsibilities 19](#__RefHeading___Toc438738855)

[b) Organize follow-up meetings 19](#__RefHeading___Toc438738856)

[7. REASSESS PRACTICE WITH FEEDBACK 20](#__RefHeading___Toc438738857)

[8. REGULARITY AUDIT OF MEETINGS 20](#__RefHeading___Toc438738858)

[9. BENEFITS OF CBCA 20](#__RefHeading___Toc438738859)

[10. DISADVANTAGES OF ACBC 21](#__RefHeading___Toc438738860)

[References 23](#__RefHeading___Toc438738861)

[Appendix 1: Form for caesarean section audit 29](#__RefHeading___Toc438738862)

[Appendix 2: Criteria for non-medically indicated caesareans 44](#__RefHeading___Toc438738863)

[Appendix 3: Analysis grid for caesarean sections audit 45](#__RefHeading___Toc438738864)

[Appendix 4: Report of the audit session 49](#__RefHeading___Toc438738865)

# Context

Since the 1970s, the rate of caesarean sections (CS) has signicantly increased worldwide (from less than 7% in the 1970s to over 25% in 2003) (WHO 1995-2003, Betrán AP and al 2007). This increasing rate has occurred without scientific evidence that high CS rates were necessarily accompanied by better maternal and perinatal outcome (Althabe F and al 2006, Betrán AP and al 2007). According to WHO recommendations, a CS rate between 5%–10% has optimal efficacy (WHO 2015). Population-level CS rates higher than10% are not associated with better health outcomes for mother and child (Villar and al 2007, Althabe F and al 2006), and are sometimes associated with negative consequences in maternal and child health (Villar and al 2007, Souza JP 2010).

The increase in CS rates is largely due to the growing practice of CS without medical reason (Souza JP and al 2010, Lavender T and al 2102). A third of the 18.5 million caesareans performed annually around the world would not be medically indicated (WHO 2010). Although middle and high income countries account for most of the rise in the trend of unnecessary CS, more studies report a similar trend in low-income countries (Aminu and al 2014).

## Caesarean section in sub-Saharan Africa

In Africa, although the population-based CS rate remains below 5% in many countries (Villar and al 2006), the existing data show that the rate of CS without medical indication in existing facilities is still as high as in Latin America or Asia (Shah A and al 2009).

The main indications for CSs in sub-Saharan Africa are: obstructed or prolonged labor, previous CS, fetal distress and pre-eclampsia / eclampsia.

In a study conducted in 46 referral hospitals in Senegal and Mali (Briand V et al 2008), the main indications of CSs reported by recipients were: dystocia or prolonged labor (29%); previous CS (18%); fetal distress (12%); preeclampsia / eclampsia (4%), retro-placental hematoma (3%).

Factors influencing the practice of non-medically indicated caesarean (NMIC) are multi-factorial and are related to clinicians, patients or characteristics of the health system. The low level of qualification of the clinician (20), the lack of use of evidence-based guidelines (Maaløe N and al 2012, Koroukian SM and al 1998), fear of lawsuits and criticism by peers (Niino Y and al 2011, Hellerstein S and al 2015) are the known factors associated with an abusive practice of CS. Social pressure and needs to plan birth (Penna L and al 2003), the perception of greater safety for the baby (Niino Y and al 2011, Hellerstein S and al 2015), the lack of information on the risks associated with CS (Dursun P and al 2011) and the fear of the pain associated with labor (Penna L and al 2003) are the main reasons why women request a CS**.** Finally, the private practice of medicine (Hopkins K and al 2013), childbirth at a teaching hospital and / or with a high number of annual delivery (Althabe F and al 2004), insufficient or lack of qualified personnel (Aminu M and al 2014), and incentives paid to claimants systems increase the risk of NMIC ( Hellerstein S and al 2015, Bogg L and al 2010).

In Burkina Faso, the population-based CS rate is still low (2%) (EDS 2010). However, CS in hospitals are steadily increasing, exceeding 40% of deliveries in some hospitals in 2013 (Annuaire statistique 2013 BF) since the implementation of emergency obstetric care subsidy policy in 2006 (Ridde V and al 2011). In this context, the decision to perform a CS may be taken by a specialist (obstetrician), but also by a general practitioner (GP), midwife or nurse trained in emergency surgery. An earlier study conducted in 10 hospitals in Burkina Faso, based on experts’ opinion, showed that the low level of qualification of the clinician was associated with a risk of NMJC multiplied by 4 compared to an obstetrician-gynecologist (Kouanda S and al 2014). Although improving access to CS is a necessity in a country where the population-based rate remains low, it would simultaneously implement a quality improvement program to prevent parallel increase in caesarean abusive.

## Interventions to reduce caesarean section rates

High rates of caesarean is a major concern because of the potential harm to the mother and baby associated with caesarean delivery without medically indication and costs related to health care (National Collaborating Centre for Women's and Children's Health 2004. Liu S et al 2005; Hall MH et al 1999; Alexander S et al 2003; Lydon-Rochelle M et al 2000; Allen VM et al 2003; Zanardo V et al 2004; Levine EM et al 2001). To provide healthcare professionals with recommendations based on scientific evidence regarding the appropriate selection of women who should benefit from the caesarean is now a priority.

Various strategies for reducing the proportion of NMICs have been tested and evaluated. Obtaining a second opinion on the indication for a caesarean before proceeding is a factor in reducing excessive rates (Althabe F and al 2004). Encouraging normal, midwife-assisted deliveries outside the hospital could also reduce NMIC rates (Briand V and al 2012, Niino Y and al 2011). Other strategies include developing clear guidelines based on the recommendations of professional associations and instructing health professionals in best practices to improve their knowledge (Saha S and al 2012, Hartmann KE and al 2012). Auditing indications for caesareans and providing feedback to health professionals, combined with instituting best practices for managing labour and performing caesareans, have resulted in significantly lower rates of caesareans among low-risk women (Chaillet N and al 2015). A meta-analysis of 10 randomized controlled trials in high-income countries showed a 19% reduction in caesarean rates. The most effective strategies were clinical audits with feedback (RR = 0.87; 95% CI = 0.81, 0.93), continuous quality improvement strategies (RR = 0.74; 95% CI = 0.70, 0.77), and multi-pronged interventions combining several approaches (RR = 0.73; 95% CI = 0.68, 0.79) (26, 27).

A systematic review of interventions in low-income countries to improve the performance of health professionals suggested that: 1) simple distribution of written directives is most often ineffective; 2) supervision and clinical audits with feedback are generally effective; and 3) complex interventions may be more effective than simple ones (Rowe AK and al 2005). Furthermore, the use of SMS (Short Message Service) technology appears to have a positive, and less costly, impact on the continuing education of healthcare providers in sub-Saharan Africa ( Cole-Lewis H and al 2010, Fjeldsoe BS and al 2009, Krishna S and al 2009 ). Indeed, studies on its use have shown SMS is easy to implement and produces positive results in terms of improved knowledge and practices among health professionals in different contexts ( Callan P and al 2011, Jones COH and al 2012).

While the results of randomized controlled trials in high-income countries are encouraging (Chaillet N and al 2015), we found no evidence that these interventions, whether alone or in combination, are effective in reducing NMIC rates in low- or middle-income countries.

The objective of the DECIDE (Appropriate decision for CS in Burkina Faso) trial is to evaluate the effectiveness and understand the implementation of an intervention combining three potentially effective approaches for reducing NMIC rates: 1) training in best practices for diagnosing the main indications for caesarean; 2) clinical audits based on objective criteria for the main indications for caesareans; and 3) SMS-based reminders to support decisions regarding clinically indicated caesareans.

***Audits are one of self-assessment mechanisms that can help maternity teams maintain or raise their level of quality of care.***

Indeed, among the various approaches to improve quality of care, clinical audit is especially recommended; a literature review conducted in 2000 involving 96 publications concludes that it can be a significant support (Johnston et al. 2000). It is also routinely used and well accepted in developed countries (Wagaarachchi et al. 2001) and increasingly tested in developing countries.

# OBJECTIVES OF THE GUIDE

This guide is drawn to the attention of health care providers of maternity services and its main objective is to contribute to improving the quality of obstetric care and the reduction of non-medically indicated caesarean.

The guide is for maternity teams that will introduce the clinical audit of CSs in their service:

• Obstetricians

• General practitioners

• Midwives and nurses

• The other staff involved in obstetric care

It is an educational tool designed to help and support these teams in their quality assessment approach to obstetric care and especially the practice of caesarean in their service; it comprises:
- A description of the different steps involved in conducting an audit, from preparation to closing a session.
-Proposals for the function of the different actors involved in the process
-Proposals for media or data sheets that are required to conduct audits, such as lists of criteria, standards of care and synthesizing sheets.

**Figure 1: Why conduct an audit? (Bailey et al. 2003)**

- Reduction in fatality rates

- Improved satisfaction of care needs

- Better use of services

Effective audit

- Best practices

- Efficient use of resources

- Improved staff motivation

#

# CLINICAL AUDITS IN GENERAL

## 1. DEFINITIONS

The term 'audit' is often used in the field of health to appoint a monitoring range of assessment methods and notification of expected results in health as well as to follow the structure or process of care (OMS 2004). As for clinical audit, it is described as a systematic and critical analysis of the quality of care in the broad sense, that is to say, including the procedures for the diagnosis, treatment, care and patients’ outcomes (Crombie et al 1997; Ronsmans 2001). This systematic review is a process that aims to improve the quality of patient care (National Institute for Clinical Excellence 2002). In this context the term "clinical" refers to the work of doctors, midwives, nurses and other health professionals.

There are several methods of audit; but there are three main approaches (Sahel Lardi, & De Brouwere 2005):

- Criteria based Audits
- The case reviews
- Confidential inquiries into deaths

Only the first approach will be discussed in this manual.

## 2. THE AUDIT CYCLE

Conventionally, an audit is carried out as a cycle consisting of five steps, steps that are repeated until the objectives are achieved.

Step 1: Establishment of good practice criteria
This step is crucial, it sets the audit topics, sets operational definitions, determines the criteria and standards of good practice and develop audit forms.

### Step 2: Measure of the current practice

The files of the cases corresponding to the chosen theme are selected to be audited.

### Step 3: Objectives to achieve

At this stage, all of the staff involved will receive and / or review the information on the results of the previous step and see if it is appropriate to make changes. For this, the objectives are to be formulated by the staff itself and an action plan to achieve them must be implemented.

**Step 1**:

Establish standard criteria of good practice

**Step 4**:

Implement practical changes

**Step 5**:

Reevaluate practice and retro-information

**Step 3**:

Render the findings and set objectives

**Step 2**:

Measure and analyze the current practice

**Figure 2: The audit cycle**

### Step 4: Changes to operate

This step puts into practice through the action plan, the changes decided and agreed in a realistic timeframe. Follow-up meetings are held regularly to ensure that agreed actions are implemented. Step 5: Reevaluate practice

Approximately one month after the definition of objectives and the start of the implementation of the actions, a new evaluation is performed and compared to the results determined in step 3. Based on the results it can be decided to repeat the action plan and start from step 2 or select other topic and repeat step 1.

## 3. PRINCIPLES OF AUDITS

Clinical audit is based on some fundamental principles:

•Research to improve practice based on the results of the audits

•Respect the of concept of practice based on scientific evidence for the establishment of norms and standards

• The process is non-punitive

• Respect for Privacy

The setting up of the audits depends on two things (Bailey et al 2003):

- The existence of standards (or protocols or treatment guidelines). Every practice or procedure should be governed by a standard. The standards used to define the criteria from which the practice will be evaluated.

- The existence of sources of information on medical practice (patient registry, well documented clinical records). The good documentation of clinical records is essential for the conduct of audits on clinical practices. If there are no records or if these poorly maintained it will be difficult to audit; "If it is not registered anywhere, it did not happen!"

## 4. INFLUENCE FACTORS OF AUDITS

### 4.1. The facilitating factors

- A minimum of resources, equipment and personnel in order to ensure minimum quality of care in the hospital.

- The commitment and support of the administrative authority to assist the team, mobilize resources and commitment for change.

- The willingness and commitment of the team that should feel concerned with improving the quality of care it provides, to be ready to challenge but that should be involved in decisions.

- A favorable environment where constructive criticism is possible and non-threatening environment.

- Support to hospital teams in methodology for the conduct of the audit by starting assistance that will enable them to acquire the knowledge necessary for the conduct of audits.

### 4.2. Obstacles

- Beliefs and a priori suspicions, doubts, fear of criticism and lack of confidentiality accented by a threatening environment or repressive

- Lack of didactic support

- Lack of resources for conducting audits

- Poor quality of the records and insufficient documentation

### 4.3. The risks

- Discourage health professionals if the proposed changes do not take place
- Encourage false reports if auditing is perceived as threatening

- Worsening relations between staff

# CRITERIA BASED CLINICAL AUDIT (CBCA)

References: Bailey et al 2003; Filippi et al. 2004; Graham et al 2003; WHO 2004; Sahel Lardi & 2005; Wagaarachchi et al. 2001

## 1. DEFINITIONS

The audit based on criteria compares the care provides to agreed criteria of optimal care. The term "criterion" refers to measurable activities that are appropriate in the context in which they are used.

The procedure will consist of extracting data from medical records of relevant cases (In this case CS), aggregation and determining the percentage of patients who received satisfactory care to the selected criteria.

From there, the process may reveal that the expected level of care is not achieved while highlighting the specific changes to be implemented in clinical practice to remedy this situation.
The effectiveness of this kind of audit will then be evaluated in terms of changes in the proportion of cases meeting the optimal criteria.

A CBCA can only be organized in a health facility where:

- There are records to find potentially interesting cases.

- There are medical charts containing information on the care provided.

CBCA do not focus on looking for errors but rather a learning process without generating a sense of guilt.

By pooling data on patients, such audit ensures the anonymity of the care provider and offers the opportunity to learn from the practice of others.

## 2. ESTABLISHMENT OF AN AUDIT TEAM

A CBCA can be implemented at any level of the health service, both in a single health facility, regardless of its size, as part of a national initiative. At the health facility, the CBCA should be initiated by the head of the department. But any health professional can initiate the process in agreement with his colleagues. The direction of the health facility must support the initiative, and the doctors, midwives, nurses, and staff responsible for keeping records must be willing to collaborate.

To carry out a CBCA, it is more common to involve a multidisciplinary team, representing different groups of service providers. The exact composition of the team will be influenced in part by the size of the health facility and also by subjects or topics chosen for the audit.
Each team member must:

- Be able to devote some of his time to this project; support and motivation of all those involved are essential

- Know and understand that the audit did not seek to blame anyone.
For example; the team could be composed of:

- Director of the hospital

- The head of the maternity department

- Doctors

- Midwives

- Nurses

- Anaesthetists

- Laboratory Technicians

- Pharmacists

- Responsible for the blood bank

- The head of the medical records

It is especially important that at least one member of the clinical staff can devote his time to conduct the audit and submit the results (assistant audits). Data collection and analysis can be performed by existing staff, for example, by those in charge of keeping.

The team will start by identifying the content of each stage of the audit cycle and establish a work schedule and responsibilities of each will be clearly marked.

## 3. SELECTION OF THEMES, OPERATIONAL DEFINITIONS AND GOOD PRACTICE CRITERIA

**(Step 1 of the audit cycle)**

The team itself will choose the problem that will be assessed, this is fundamental; it can be clinical, organizational and management or even affect the fundamental rights of patients. Similarly, for some kind of problem, various aspects may be selected. As for the evaluation of indications for CSs, the identification of the subject of audit can be based on criteria as the most frequent indications. This step will therefore fix the audit themes, establish operational definitions, determine the criteria of good practice and develop audit forms.

As part of this project, audits’ themes will concern caesarean indications; but it is important to remember that regardless of whatever the chosen element of care, the steps of the audit process always remain the same.

### a) Select a topic audit

In this research project we selected the four main caesarean indications encountered in Burkina Faso:

• The previous CS

• The prolonged / obstructed labor

• Pre-eclampsia / eclampsia

• Fetal distress

 It is fundamental that the caesarean indications can be clearly identified from the records and clinical charts.

### b) Establish operational definitions

We have defined four main CS indications.

The previous CS: the presence of a caesarean scar (patient having already undergone a CS in a previous pregnancy).

**Pre-eclampsia / eclampsia: preeclampsia** is defined as a blood pressure greater than 160 / 90mmHg after 22 weeks of gestation associated with proteinuria.

**Eclampsia** is defined by the appearance of seizures in the context of pre-eclampsia.
Prolonged or obstructed labor: any anomaly called dystocia in the progress of the delivery that can be dynamic or mechanical origin; the labor is considered as prolonged if it has duration greater than 12 hours or active phase greater than 6 hours.

**Fetal distress**: it is an unexpected alteration of fetal condition characterized by slower (less than 120 beats per minute) or acceleration (greater than 160) of the fetal heart rate and the presence of meconium in the amniotic fluid in latency, active or expulsion phase.

### c) Identify good practice criteria

The CBCA requires that a comparison be made between current practices and documented standards.

In this case, the audit concerns the relevance of caesarean indications compared to medically justified criteria.

The 'criteria' are standard developed to identify CS whose indication is not medically justified.

Regardless of how the criteria are used by the audit team, they must imperatively:
- Be based on scientific evidence

- Be measurable (audited) based on patient medical records

- Be realistic, taking into account the capabilities of the health facility in terms of staff and resources.
As part of this project, we conducted a literature review to discern the diagnostic reasoning underlying evidence-based indications for caesareans. The four main indications for caesareans in Burkina Faso are: previous caesarean, prolonged/obstructed labour, pre-eclampsia, and acute foetal distress. The relevant references were consulted and used to generate a provisional list of good practice criteria. Preference was given to evidence obtained through randomized controlled trials, considered the most rigorous. The provisional list of criteria was sent to 16 international and national experts. The experts gave their opinions on the relevance of each criterion and proposed others. The criteria retained were those validated by at least two-thirds of the experts.

The list of criteria used in this trial is in Appendix 2 of the manual. The list is specific to each indication.

### d) Identify sources of information

Data can be extracted from one or more of these sources:

- Hospital Records (inlet-outlet, death, operating room, laboratory, maternity, delivery room, intensive care)

- Patients’ medical charts

- Interview with staff and families

- Interview with patients

- Lists of pharmaceutical stocks

- Inventory of blood banks

### e) Develop structured audit forms

Once the definitions and criteria of good practice agreed, audit structured forms (forms for data extraction) for each theme must be developed, tested in advance, revised and finalized by the audit team.

The audit assistants, which can for example be the staff responsible for the maintenance of medical records, must be trained in the extraction of information from the patient records. This information shows whether the care provided met the criteria of good practice.
A data extraction form was developed as part of the DECIDE trial (Appendix 1) and will be used for this study.

The form includes the following section:

- Information on the conditions of admission of the patient

- Socio-demographic characteristics of the patient

- Medical and obstetrical history

- Information regarding the current pregnancy

- The obstetrical examination before the decision to caesarean

- Monitoring of labor

- The indication of CS

- Post-operative monitoring

## 4. ANALYSIS OF THE CURRENT PRACTICE

 (Step 2 of the audit cycle)

This step should provide a baseline assessment of current practice and is done by the analysis of patients’ medical records, in this case those who will have caesarean section for one of the four main indication retained in step 1.

### a) The number of cases

The number of cases required to produce useful results is difficult. As part of this research, we recommend that audit meetings are held monthly. The number of cases to be audited will vary depending on the size and the type of health facility concerned.

### b) Identification and retrieval of medical records

Before starting the evaluation of practice, audit assistant must check all possible sources of identification, such as registers of admission, delivery, operating, etc. It is important to use a systematic approach to identify these sources to avoid losing the case or to count some twice.

You cannot have an accurate assessment of the practice if all cases that arose during a period have not been identified. When cases are too numerous to allow to check all files, it is possible to consider a representative sample. This requires though that all eligible cases are extracts from the registry to pull-out a sample.

Caesareans performed for the four indications retained may be identified prospectively or retrospectively:

- In the case of a retrospective identification, all cases that occurred during a previous time period (usually one month) must be found.

- In the case of prospective identification, audit team regularly analyze medical records (ideally every morning when staff) to identify cases of CSs performed for each of the four selected indications.

The advantage of a prospective approach is that the chances of finding patients records are generally better than in the retrospective approach. This is the approach we recommend.

### c) Data extraction from medical records

As part of this project, this is to select among all CSs performed over a given period, those performed for the four indications retained.

Data are recorded on the audit form (Appendix 1) by the audit’s assistant who must be supervised by a senior member of staff for quality control.

In this phase, all required data may not be included in medical records. When questioning health professionals, they will state that the procedure in question has been applied, but was not recorded. This highlights poor record keeping. It should be noted that, with regard to clinical audit, one must consider that a practice or procedure that was not recorded, was not applied. Attracting the attention of staff in this procedure often causes better record keeping.

### d) Data Analysis

The analysis will be done in an auditing session in two stages: (i) identification of cases of non-medically indicated CSs and (ii) identification of the causes of non-medically indicated caesareans.

A first analysis focuses on the application of non-medically indicated caesarean criteria. The presentation is done in tabular form (Table1 is an example of reporting results).

**TABLE 1: Example of presentation of data analysis results**

| Indication | Total number of caesareans | Number of non-medically indicated caesareans | % of non-medically indicated caesareans |
| --- | --- | --- | --- |
| Prolonged / obstructed labor | 508 | 107 | **21.01%** |
| Pre-eclampsia/eclampsia | 133 | 26 | **19.54%** |
| Previous caeserean section | 378 | 69 | **18.25%** |
| Fœtal distress | 670 | 196 | **29.25%** |
| **Total** | 1689 | 398 | **23.56%** |

In the next step of the analysis, non-medically indicated caesareans are selected to identify the causes. At this stage, it is recommended to use the analytical framework presented in appendix3 of this guide. This grid provides a directory of key questions to identify medical and non-medical items (including organizational) that have to be enhanced or modified in current practice to reduce the number of non-medically indicated caesarean.

The results of the data analysis are recorded at the end of the session in the audit report presented in appendix4.

The audit report is a synthesis of audit activities in which are notified:

- The proportion of non-medically indicated CSs for each of the four caesarean indications

-The overall proportion of non-medically indicated caesareans for the four caesarean indications

- The key issues identified in the management of cases audited

- The positives points in the management of cases audited

- The main causes of dysfunction identified

- The solutions to overcome the identified causes,

- The responsible identified for the implementation of solutions and agreed deadlines.

Audit report tracks the implementation of agreed recommendations. It is a repository for the revaluation of practice.

## 5. CONCLUSIONS AND OPPORTUNITY TO SET OBJECTIVES

**(Step 3 of the audit cycle)**

At this stage, all staff involved in obstetric care receives a summary of the results of the evaluation of current practice.

Staff is invited to estimate how the criteria have been met; then the reality is established with the percentage of non-medically caesareans and the elements that can be improved.
The percentage of non-medically indicated caesarean ranges from xx% to xx% depending on the indication and this may be an impetus for change.

It is also necessary ensure counterbalance the deficiencies highlighted by drawing attention to good practices followed.

The objective is that the staff themselves fixed the percentage of non-medically indicated caesareans for the next evaluation. If staff is actively involved in the development of objectives, it is more likely to implement changes and he appropriates the audit procedure.

### Develop an action plan

After the identification of areas for improvement, the audit team establishes an action plan with the help of experienced clinicians.

It is necessary that the actions envisaged are trying to meet the causes of the deficiencies that have been identified by staff.

Deficiencies may concern:

- Organization of care

- Insufficient knowledge and / or inadequate

- Insufficient skills and / or inappropriate

- Inappropriate attitudes

The proposed changes must be realistic, affordable and sustainable to meet the objectives set by the staff themselves. These changes may concern organization of training sessions for staff, improving the ranking of medical records or ensuring compliance with the protocols.

## 6. IMPLEMENTATION OF THE PLAN OF ACTION

(Step 4 of the audit cycle)

The necessary changes are made via the action plan.

### a) Assign roles and responsibilities

It is essential to identify the people who will be responsible for the implementation and the evaluation of agreed activities and within the agreed deadlines.

### b) Organize follow-up meetings

Follow-up meetings should be held regularly to ensure that agreed actions are implemented, that the action plan is feasible or requires adaptations.

Moreover, these meetings can help:

- Maintain motivation and enhance mutual willingness for change.

- To increase the chances that the changes are maintained over time.

## 7. REASSESS PRACTICE WITH FEEDBACK

(Step 5 of the audit cycle)

The audit cycle ends with a new evaluation of the practice of CS based on the selected criteria. The results are compared with the objectives determined in step 3. Based on the results it can be decided to repeat the action plan or select other goals. It is also possible to select a new theme and thus repeating the cycle from step 1.

## 8. REGULARITY AUDIT OF MEETINGS

As part of this project, we recommend that the audit meetings are held monthly and that all caesareans performed for the four indications are included each time. For caesareans performed for others indications than those retained, the rationality of the indication cannot be assessed but can nevertheless appreciate the relevance to implicit criteria defined by the senior of the staff.

## 9. BENEFITS OF CBCA

- **The participatory element**.

Involve staff in the process of reflection on practice and the development of the objectives is probably a care quality improvement mechanism

- **Educational Tool.**

The audit is an excellent educational tool which, when carried out according to the rules, is not repressive.

**- Feedback.**

The audit can provide direct feedback to those concerned about the performance of their practices. The participatory process will also enable them to identify realistic solutions to improve them.

**- Efficiency**

Data extraction and analysis can be performed by non-medical staff, which can be a cheaper form of audit.

- **Quick Passage to action.**

The audit can be implemented locally and generate locally relevant and immediately usable information.

**- Objective Evaluation.**

The audit process creates a structured framework for data collection, leaving less space to subjective evaluation of the practices.

- **Secondary benefit.**

Auditing can also highlight deficiencies in the documentation and archiving of clinical records.

## 10. DISADVANTAGES OF ACBC

- **The community's opinion is not taken into account.**

The audit is limited to the evaluation of clinical care in a well-defined health structure. It is therefore not interested in the problems that exist in the community.

- **Little known concepts of health professionals.**

Some clinicians are used to relying on their own experience and their preferences. Therefore, they may find that the concept of practice based on scientific evidence and that of an audit are not only quite difficult to understand but also felt as a threat to their practice and reputation. Hence the importance to organize, before the establishment of an audit process, a seminar to allow clinicians to become familiar with these concepts and to reassure them.

- **Difficulty in developing criteria.**

Appropriate criteria should be, or available or be created.

-**Dependence on the quality of the existing information system.**

The audit requires, to be feasible, documentation and information that must be available in the medical records.

**- Limited in themes**.

The audit can only handle certain issues and therefore do not provide a complete overview of the management of patients.

- **Time Consumer.**

Audit assistants, responsible for finding medical charts, extract the data and present the results, should be available.

# References

***World Health Organization: CDS INAS Bulletins 1995 – 2003. WHO;***

***Betrán AP, Merialdi M, Lauer JA, Bing-Shun W, Thomas J, Van Look P, et al. Rates of caesarean section: analysis of global, regional and national estimates. Paediatr Perinat Epidemiol. mars 2007;21(2):98‑113.***

***Althabe F, Sosa C, Belizán JM, Gibbons L, Jacquerioz F, Bergel E. Cesarean section rates and maternal and neonatal mortality in low-, medium-, and high-income countries: an ecological study. Birth Berkeley Calif. déc 2006;33(4):270‑7.***

***Villar J, Valladares E, Wojdyla D, Zavaleta N, Carroli G, Velazco A, et al. Caesarean delivery rates and pregnancy outcomes: the 2005 WHO global survey on maternal and perinatal health in Latin America. Lancet Lond Engl. 3 juin 2006;367(9525):1819‑29***

***OMS. Déclaration de l’OMS sur les taux de césarienne. 2015.***

***Souza JP, Gülmezoglu A, Lumbiganon P, Laopaiboon M, Carroli G, Fawole B, et al. Caesarean section without medical indications is associated with an increased risk of adverse short-term maternal outcomes: the 2004-2008 WHO Global Survey on Maternal and Perinatal Health. BMC Med. 2010;8:71.***

***Lavender T, Hofmeyr GJ, Neilson JP, Kingdon C, Gyte GML. Caesarean section for non-medical reasons at term. Cochrane Database Syst Rev. 2012;3:CD004660.***

***WHO. World Health Organization: The global numbers and costs of additionally needed and unnecessary caesarean sections performed per year: overuse as a barrier to universal coverage. 2010, http://www.who.int/ healthsystems/topics/financing/healthreport/30C-sectioncosts.pdf). 2010.***

***Aminu M, Utz B, Halim A, van den Broek N. Reasons for performing a caesarean section in public hospitals in rural Bangladesh. BMC Pregnancy Childbirth. 2014;14:130.***

***Shah A, Fawole B, M’imunya JM, Amokrane F, Nafiou I, Wolomby J-J, et al. Cesarean delivery outcomes from the WHO global survey on maternal and perinatal health in Africa. Int J Gynaecol***

***Briand V, Dumont A, Abrahamowicz M, Traore M, Watier L, Fournier P. Individual and institutional determinants of caesarean section in referral hospitals in Senegal and Mali: a cross-sectional epidemiological survey. BMC Pregnancy Childbirth. 2012;12:114.***

***Koroukian SM, Trisel B, Rimm AA. Estimating the proportion of unnecessary Cesarean sections in Ohio using birth certificate data. J Clin Epidemiol. déc 1998;51(12):1327‑34. Obstet Off Organ Int Fed Gynaecol Obstet. déc 2009;107(3):191‑7.***

***Maaløe N, Sorensen BL, Onesmo R, Secher NJ, Bygbjerg IC. Prolonged labour as indication for emergency caesarean section: a quality assurance analysis by criterion-based audit at two Tanzanian rural hospitals. BJOG Int J Obstet Gynaecol. avr 2012;119(5):605‑13.***

***Niino Y. The increasing cesarean rate globally and what we can do about it. Biosci Trends. août 2011;5(4):139‑50.***

***Hellerstein S, Feldman S, Duan T. China’s 50% caesarean delivery rate: is it too high? BJOG Int J Obstet Gynaecol. janv 2015;122(2):160‑4.***

***Dursun P, Yanik FB, Zeyneloglu HB, Baser E, Kuscu E, Ayhan A. Why women request cesarean section without medical indication? J Matern-Fetal Neonatal Med Off J Eur Assoc Perinat Med Fed Asia Ocean Perinat Soc Int Soc Perinat Obstet. sept 2011;24(9):1133‑7***

***Penna L, Arulkumaran S. Cesarean section for non-medical reasons. Int J Gynaecol Obstet Off Organ Int Fed Gynaecol Obstet. sept 2003;82(3):399‑409.***

***Hopkins K, de Lima Amaral EF, Mourão ANM. The impact of payment source and hospital type on rising cesarean section rates in Brazil, 1998 to 2008. Birth Berkeley Calif. juin 2014;41(2):169‑77***

***Althabe F, Belizán JM, Villar J, Alexander S, Bergel E, Ramos S, et al. Mandatory second opinion to reduce rates of unnecessary caesarean sections in Latin America: a cluster randomised controlled trial. Lancet Lond Engl. 12 juin 2004;363(9425):1934‑40***

***Bogg L, Huang K, Long Q, Shen Y, Hemminki E. Dramatic increase of Cesarean deliveries in the midst of health reforms in rural China. Soc Sci Med 1982. mai 2010;70(10):1544‑9.***

***Institut National de la Statistique et de la Démographie (INSD) Ministère de l’Économie et des Finances Ouagadougou, Burkina Faso. BURKINA FASO Enquête Démographique et de Santé et à Indicateurs Multiples (EDSBF-MICS IV) 2010.***

***Direction générale des études et des statistiques sectorielles, UNICEF. ANNUAIRE STATISTIQUE 20 13***

***Ridde V, Richard F, Bicaba A, Queuille L, Conombo G. The national subsidy for deliveries and emergency obstetric care in Burkina Faso. Health Policy Plan. nov 2011***

***Kouanda S, Coulibaly A, Ouedraogo A, Millogo T, Meda BI, Dumont A. Audit of cesarean delivery in Burkina Faso. Int J Gynaecol Obstet Off Organ Int Fed Gynaecol Obstet. juin 2014;125(3):214‑8.***

***Khawaja M, Jurdi R, Kabakian-Khasholian T. Rising trends in caesarean section rates in Egypt. Birth Berkeley Calif. 2004 Mar;31(1):12–6.***

***World Health Organization. Beyond the numbers : reviewing maternal deaths and complications to make pregnancy safer. World Health Organization, Geneva; 2004.***

***Dumont A, M.Chergui, Alioune Gaye, Anne Landry, C.Tourigny, Pierre Fournier. Identifying barriers and facilitators towards implementing facility-based maternal death reviews in Senegal. Int Womens Health Spec Rep J Obstet Gynaecol. 2008;***

***Dumont A, Gaye A, de Bernis L, Chaillet N, Landry A, Delage J, et al. Facility-based maternal death reviews: effects on maternal mortality in a district hospital in Senegal. Bull World Health Organ. 2006 Mar;84(3):218–24.***

***Dumont A, de Bernis L, Bouvier-Colle MH, Bréart G, MOMA study group. Caesarean section rate for maternal indication in sub-Saharan Africa: a systematic review. Lancet. 2001 Oct 20;358(9290):1328–33.***

***Fesseha N, Getachew A, Hiluf M, Gebrehiwot Y, Bailey P. A national review of caesarean delivery in Ethiopia. Int J Gynaecol Obstet Off Organ Int Fed Gynaecol Obstet. 2011 Oct;115(1):106–11.***

***Runmei M, Terence T L, Yonghu S, Hong X, Yuqin T, Bailuan L, et al. Practice audits to reduce caesareans in a tertiary referral hospital in south-western China. Bull World Health Organ. 2012 Jul 1;90(7):488–94.***

***Abou-Zahr C, Wardlaw T. The World Health Report 2005 - make every mother and child count. World Health Organization; 2005.***

***Rowe AK, de Savigny D, Lanata CF, Victora CG. How can we achieve and maintain high-quality performance of health workers in low-resource settings? Lancet. 2005 Sep 17;366(9490):1026–35.***

***Marchal B,, Kegels G. Health workforce imbalances in times of globalization: brain drain or professional mobility? Int J Health Plann Manage. 2003 Dec;18(Suppl1):S89–101.***

***Kouanda S, Coulibaly A, Ouedraogo A, Millogo T, Meda BI, Dumont A. Audit of caesarean delivery in Burkina Faso. Int J Gynaecol Obstet Off Organ Int Fed Gynaecol Obstet. 2014 Jun;125(3):214–8.***

***National Collaborating Centre for Women’s and Children’s Health. Caesarean section: clinical guideline. London: RCOG Press, 2004.***

***Liu S, Heaman M, Joseph KS, et al. Risk of maternal postpartum readmission associated with mode of delivery. Obstet Gynecol 2005; 105: 836-42.***

***Hall MH, Bewley S. Maternal mortality and mode of delivery. Lancet 1999; 354:776.***

***Alexander S, Wildman K, Zhang W, Langer M, Vutuc C, Lindmark G. Maternal health outcomes in Europe. Eur J Obstet Gynecol Reprod Biol 2003; 111: Suppl 1:S78-S87.***

***Lydon-Rochelle M, Holt VL, Martin DP, Easterling TR. Association between method of delivery and maternal rehospitalization. JAMA 2000; 283: 2411-6.***

***Allen VM, O’Connell CM, Liston RM, Baskett TF. Maternal morbidity associated with caesarean delivery without labor compared with spontaneous onset of labor at term. Obstet Gynecol 2003; 102: 477-82.***

***Zanardo V, Simbi AK, Franzoi M, Soldà G, Salvadori A, Trevisanuto D. Neonatal respiratory morbidity risk and mode of delivery at term: influence of timing of elective caesarean delivery. Acta Paediatr 2004; 93: 643-7.***

***Levine EM, Ghai V, Barton JJ, Strom CM. Mode of delivery and risk of respiratory diseases in newborns. Obstet Gynecol 2001; 97: 439-42.***

***Hartmann KE, Andrews JC, Jerome RN, Lewis RM, Likis FE, McKoy JN, et al. Strategies to reduce cesarean birth in low-risk women [Internet]. Rockville, MD: Agency for Healthcare Research and Quality (US); 2012 [cited 2015 May 1]. Available from: http://www.ncbi.nlm.nih.gov/books/NBK114747/***

***Chaillet N, Dumont A, Abrahamowicz M, Pasquier J-C, Audibert F, Monnier P, et al. A cluster-randomized trial to reduce cesarean delivery rates in Quebec. N Engl J Med. 2015;372(18):1710–21.***

***Chaillet N, Dumont A. Evidence-based strategies for reducing cesarean section rates: a meta-analysis. Birth. 2007;34(1):53–64.***

***Rowe AK, De Savigny D, Lanata CF, Victora CG. How can we achieve and maintain high-quality performance of health workers in low-resource settings? Lancet. 2005;366(9490):1026–35.***

***Cole-Lewis H, Kershaw T. Text messaging as a tool for behavior change in disease prevention and management. Epidemiol Rev. 2010;32(1):56–69.***

***Fjeldsoe BS, Marshall AL, Miller YD. Behavior change interventions delivered by mobile telephone short-message service. Am J Prev Med. 2009;36(2):165–73.***

***Krishna S, Boren SA, Balas EA. Healthcare via cell phones: a systematic review. Telemed J E Health. 2009;15(3):231–40.***

***Callan P, Miller R, Sithole R, Daggett M, Altman D, O'Byrne D. Harnessing the mobile revolution to bridge the health education and training gap in developing countries. iHeed Institute. 2011. Available from: http://mhealthknowledge.org/resources/mhealth-education***

***Jones COH, Wasunna B, Sudoi R, Githinji S, Snow RW, Zurovac D. “Even if you know everything you can forget”: health worker perceptions of mobile phone text-messaging to improve malaria case-management in Kenya. PLoS One 2012;7(6):e38636.***

***Zurovac D, Sudoi RK, Akhwale WS, Ndiritu M, Hamer DH, Rowe AK, et al. The effect of mobile phone text-message reminders on Kenyan health workers’ adherence to malaria treatment guidelines: a cluster randomised trial. Lancet. 2011;378(9793):795–803.***

***Black RS, Brocklehurst P. A systematic review of training in acute obstetric emergencies. BJOG. 2003;110(9):837–41.***

***Dumont A, Chergui M. Gaye A, Landry A, Tourigny C, Fournier P. Identifying barriers and facilitators towards implementing facility-based maternal death reviews in Senegal. The International Women's Health Program of the SOGC: Special Report; 2008:22–27.***

***Grant A, Treweek S, Dreischulte T, Foy R, Guthrie B. Process evaluations for cluster-randomised trials of complex interventions: a proposed framework for design and reporting. Trials. 2013;14:15.***

***Moore GF, Audrey S, Barker M, Bond L, Bonell C, Hardeman W, et al. Process evaluation of complex interventions: Medical Research Council guidance. BMJ. 2015;350:h1258.***

***Bailey, P., Fortney, J. A., Freedman, L., Goodburn, E. A., Kwast, B. E., Mavalankar,D., & Moneypenny, B. 2003, Améliorer les Soins Obstétriques d'Urgence grâce à l'Audit reposant sur des Critères, Mailman School of Public Health, Columbia University.***

***Crombie, I. E., Davies, H. T., Abraham, S. C., & Florey, C. d. V. 1997, The audit handbook. Improving health care through clinical audit John Wiley & Sons, Chichester, England.***

***Dumont, A., Gaye, A., de Bernis, L., Chaillet, N., Landry, A., Delage, J., & Bouvier- Colle, M. H. 2006, "Facility-based maternal death reviews: effects on maternal mortality in a district hospital in Senegal", Bulletin of the World Health Organisation,vol. 84, no. 3, pp. 218-224.***

***Filippi, V., Brugha, R., Browne, E., Gohou, V., Bacci, A., De Brouwere, V., Sahel, A., Goufodji, S., Alihonou, E., & Ronsmans, C. 2004, "Obstetric audit in resourcepoor settings: lessons from a multi-country project auditing 'near miss' obstetrical emergencies", Health Policy Plan., vol. 19, no. 1, pp. 57-66.***

***Graham, W., Bullough, C., & Graham, P. 2003, Une Introduction à l'Audit Clinique Basé sur des Critères (ACBC) par Formation Assistée par Ordinateur, Université d’Aberdeen.***

***Graham, W., Wagaarachchi, P., Penney, G., McCaw-Binns, A., Antwi, K. Y., & Hall, M. H. 2000, "Criteria for clinical audit of the quality of hospital-based obstetric care in developing countries", Bulletin of the World Health Organization, vol. 78, no. 5, pp. 614-620.***

***Johnston, G., Crombie, I. K., Davies, H. T. O., Alder, E. M., & Millard, A. 2000, "Reviewing audit: barriers and facilitating factors for effective clinical audit", Quality in Health Care, vol. 9, no. 1, pp. 23-36. Lewis, G. 2003, "Beyond the numbers: reviewing maternal deaths and complications to make pregnancy safer", Br.Med Bull., vol. 67, pp. 27-37.***

***Althabe F, Belizán JM, Villar J, Alexander S, Bergel E, Ramos S, et al. Mandatory second opinion to reduce rates of unnecessary caesarean sections in Latin America: a cluster randomised controlled trial. Lancet. 2004 Jun 12;363(9425):1934–40.***

***National Institute for Clinical Excellence 2002, Principles for Best Practice in Clinical Audit Radcliffe Medical Press Ltd, Abingdon.***

***OMS 2004, Au-delà des nombres - Examiner les morts maternelles et les complications pour réduire les risques liés à la grossesse OMS, Genève.***

***Ronsmans, C. 2001, "Les audits peuvent-ils améliorer la qualité des soins obstétricaux?," in Réduire les risques de la maternité : stratégies et evidence scientifique, V. de Brouwere & W. Van Lerberghe, eds., ITGPress, Antwerp, pp. 217-238.***

***Sahel, A. & Lardi, M. 2005, Guide d'audit clinique; Un guide à l'usage des équipes des maternités hospitalières, Ministère de la Santé, Royaume du Maroc.***

***Wagaarachchi, P., Asare, K., Ashley, D., Gordon, G., Graham, W., Hall, M., Henneh, R., McCaw-Binns, A., Penney, G., Yeboah Antwi, K., & Bullough, C. 2001a, Improving the quality of obstetric care in developing countries through criterion based clinical Audit - A practical field guide Dugald Baird Centre for Research on Women's Health, Aberdeen.***

***Wagaarachchi, P. T., Graham, W. J., Penney, G. C., McCaw-Binns, A., Antwi, K. Y., & Hall, M. H. 2001b, "Holding up a mirror: changing obstetric practice through criterion-based clinical audit in developing countries", International Journal of Gynecology & Obstetrics, vol. 74, no. 2, pp. 119-130.***

# Appendix 1: Form for caesarean section audit

| **Variable** | **Question** | **Code** | **Jump** |
| --- | --- | --- | --- |
| FA01 | Hospital name | RH Dédougou = 001  MCS Solenzo = 002  RH Banfora = 003  MCS Bogodogo = 004  MCS Boulmiougou = 005  MCS Nongr-Massom= 006  RH Tenkodogo = 007  MCS Koupéla = 008  RH Kaya = 009  MCS Boulsa = 010  RH Koudougou = 011  RH Fada = 012  MCS Do = 013  MCS Dafra = 014  MCS Houndé = 015  MCS Orodara = 016  RH Ouahigouya = 017  MCS Yako = 018  MCS Zorgho = 019  RH Dori = 020  MCS Djibo = 021  RH Gaoua = 022 |  |
| FA02 | Patient name | (Open field) |  |
| FA03 | Patient surname | (Open field) |  |
| FA04 | Patient age (years) | (numeric) |__|__|years |  |
| FA05 | Lieu de résidence de la patiente | Rural = 1  Urban parceled = 2  Urban non parceled = 3 |  |
| FA06 | Name district / area / village patient | (Open field) |  |
| FA07 | Patient phone | (numeric) |  |
| FA08 | Patient weight beginning pregnancy | (numeric)|__|__|__|Kg |  |
| FA09 | Patient weight ending pregnancy | (numeric)|__|__|__|Kg |  |
| FA10 | Name provider indicated caesarean section | (a list will be proposed for each hospital after the inclusion visit)  |__|__|__| see codes |  |
| FA11 | Surname provider indicated caesarean section | (a list will be proposed for each hospital after the inclusion visit) |  |
| FA12 | Name provider has realized the caesarean | (a list will be proposed for each hospital after the inclusion visit)  |__|__|__| see codes |  |
| FA13 | Surame provider who realized the caesarean | (a list will be proposed for each hospital after the inclusion visit) |  |
| FA14 | Date admission | (dd/mm/yyyy) |  |
| FA15 | Time admission | (hh:mm) |  |
| FA16 | Woman admitted after a reference? | no=0, yes=1 | If no go to FA22 |
| FA17 | Type health facility who referred the woman | Primary health center = 1  District hospital = 2  Regional hospital = 3  Other = 6 |  |
| FA18 | Name health facility who referred the woman | (a list will be proposed for each hospital after the inclusion visit) |  |
| FA19 | Distance (km) between hospital who made the referral and this hospital | (numeric)|__|__|__| Km |  |
| FA20 | Reason (s) Reference  Many possible responses  - Labour on term pregnancy  - Labour on scarred uterus  - engagement failure at full dilation  - Failure of induction  - Retention of the second twin  - Vicious Presentation  - Abnormalities of amniotic fluid  - Precious Pregnancy  - Premature rupture of membranes  - Premature delivery threat  - Hypertension in pregnancy  - Eclampsia / HELLP syndrome  - Maternal Pathology on pregnancy  - Anaemia in pregnancy  - Pregnancy endded / not active or intrauterine fetal death (IUFD) or retention of death egg (ROM)  - Post-term pregnancy  - Malaria on pregnancy  - Cephalopelvic disproportion  - Fetal distress  - Pathological / limit / unbalanced / narrowed / immature pelvis  - Placenta previa  - Fetal macrosomia  - Pre-rupture / uterine rupture  - Vaginal bleeding at the end of pregnancy  - Urinary tract infection during pregnancy  - Placental abruption  - Vulvar / perineal condylomata  - Multiple pregnancy  - HIV on pregnancy  - Cord Abnormalities (prolapsed cord / cord circular)  - Other | (series of click)  |__|  |__|  |__|  |__|  |__|  |__|  |__|  |__|  |__|  |__|  |__|  |__|  |__|  |__|  |__|  |__|  |__|  |__|  |__|  |__|  |__|  |__|  |__|  |__|  |__|  |__|  |__|  |__|  |__|  |__|  |__| |  |
| FA21 | If other, specify | (Open field) |  |
| FA22 | Reason for admission  Many possible responses   - - Labour on term pregnancy - - Labour on scarred uterus - - engagement failure at full dilation - - Failure of induction - - Retention of the second twin - - Vicious Presentation - - Abnormalities of amniotic fluid - - Precious Pregnancy - - Premature rupture of membranes - - Premature delivery threat - - Hypertension in pregnancy - - Eclampsia / HELLP syndrome - - Maternal Pathology on pregnancy - - Anaemia in pregnancy - - Pregnancy endded / not active or intrauterine fetal death (IUFD) or retention of death egg (ROM) - - Post-term pregnancy - - Malaria on pregnancy - - Cephalopelvic disproportion - - Fetal distress - - Pathological / limit / unbalanced / narrowed / immature pelvis - - Placenta previa - - Fetal macrosomia - - Pre-rupture / uterine rupture - - Vaginal bleeding at the end of pregnancy - - Urinary tract infection during pregnancy - - Placental abruption - - Vulvar / perineal condylomata - - Multiple pregnancy - - HIV on pregnancy - - Cord Abnormalities (prolapsed cord / cord circular) - - Other | (series of click)  |__|  |__|  |__|  |__|  |__|  |__|  |__|  |__|  |__|  |__|  |__|  |__|  |__|  |__|  |__|  |__|  |__|  |__|  |__|  |__|  |__|  |__|  |__|  |__|  |__|  |__|  |__|  |__|  |__|  |__|  |__| |  |
| FA23 | If other, specify | (Open field) |  |
| **Socio-economic characteristics** | | | |
| FA24 | Patient education level | Unschooled illiterate = 0  Literate but not schooled = 1  Primary level = 2  Lower secondary = 3  Upper secondary school = 4  Higher educational level = 5 |  |
| FA25 | Spouse education Level | Unschooled illiterate = 0  Literate but not schooled = 1  Primary level = 2  Lower secondary = 3  Upper secondary school = 4  Higher educational level = 5 |  |
| FA26 | Patient profession | Housewife 1 =  Breeder / farmer = 2  Trader = 3  Employee = 4  Private employee = 5  Other = 6 | If other, go toFA27 If no go to FA28 |
| FA27 | If other, specify | (Open field) |  |
| FA28 | Spouse profession | Breeder / farmer =1  Trader = 2  Employee = 3  Private employee = 4    Other = 6 | If other, go toFA29 If no, go to FA30 |
| FA29 | If other, specify | (Open field) |  |
| FA30 | Total amount paid for drugs, consumables and other products paid in the HF where was held caesarean (CFA) | (numeric)  |__|__|__|__|__|CFA |  |
| FA31 | Total amount paid for acts of care in the HF where was held caesarean (CFA) | (numeric)  |__|__|__|__|__| CFA |  |
| FA32 | Total amount paid for transportation between the HF and who referred the hospital where they underwent caesarean delivery (CFA)? | not applicable= 88 888 (if FA16 = no)  |__|__|__|__|__| CFA |  |
| FA33 | Amount for hospitalization (CFA) | |__|__|__|__|__| CFA |  |
| **Possession of household property** | | | |
| FA34 | Does the household own a motorcycle? | no=0, yes=1 |  |
| FA35 | The household does have a car? | no=0, yes=1 |  |
| FA36 | Does the household own a fixed telephone? | no=0, yes=1 |  |
| FA37 | How many mobile phones does the household have?  (If no mobile phones put 0) | (numeric) |__| |  |
| FA38 | How many hens / Guinea fowl / turkey / duck does the household has?  (If no animals put 0) | (numeric)  |__|__|__|__| |  |
| FA39 | How many sheep / goats / pigs does the household have?  (If no animals put 0) | (numeric)  |__|__|__|__| |  |
| FA40 | How many cattle / camel / donkey does the household have?  (If no animals put 0) | (numeric)  |__|__|__|__| |  |
| FA41 | Does the household have drinking water? | no=0, yes=1 |  |
| FA42 | Type of household housing | Homeowner = 1  Rental housing = 2 |  |
| FA43 | Type the household dwelling | Made of cement = 1  Mudbrick = 2  Mixed = 3 |  |
| FA44 | Does the household have air conditioner ? | no=0, yes=1 |  |
| **Clinical history** | | | |
| FA45 | Medical  Many possible responses     - HIV - Diabetes - High blood pressure - Sickle Cell Disease - Asthma - Peptic ulcer - Heart Disease - Hepatitis - Tuberculosis - Toxoplasmosis - Rubella - Genital infection - Syphilis - Other diseases | (series of click)  *|__|*  *|__|*  *|__|*  |__|  *|__|*  *|__|*  *|__|*  *|__|*  *|__|*  *|__|*  *|__|*  *|__|*  *|__|*  *|__|* | If other, go toFA46 If no, go to FA47 |
| FA46 | If other pathology, specify | (Open field) |  |
|  | **Obstetric** |  |  |
| FA47 | Number of pregnancies | (numeric)  |__|__| | If 01 go to FA 56 |
| FA48 | Number of deliveries | (numeric)  |__|__| |  |
| FA49 | Number of previous abortions | (numeric)  |__|__| |  |
| FA50 | Number of previous caesarean | (numeric)  |__|__| | If 00 go to FA53 |
| FA51 | History of corporeal caesarean section? | Yes=1,  no = 0  not documented = 9 |  |
| FA52 | Date last caesarean | (dd/mm/yyyy)  not documented=09 09 9999 |  |
| FA53 | Date last birth | (dd/mm/yyyy)  not documented=09 09 9999 |  |
| FA54 | Other obstetric history | no=0, yes=1 | If no, go to  FA56 |
| FA55 | If other obstetric history, specify: | (Open field) |  |
|  | **Surgical** |  |  |
| FA56 | Surgical history of the patient  Many possible responses  - Polymyomectomie  - Cure of uterine malformation  - Cure of obstetric fistula  - Appendectomy  - Salpingectomy  - oophorectomy  - Peritonitis  - Bowel obstruction  - Conization / cryotherapy  - Cure of synechia  - Pelvic surgery  - Pelvic fracture  - Other | (series of click)  *|__|*  *|__|*  *|__|*  *|__|*  *|__|*  *|__|*  *|__|*  *|__|*  *|__|*  *|__|*  *|__|*  *|__|*  *|__|* | If other, go to FA57 If no, go to FA58 |
| FA57 | If other surgical history, specify | (Open field) |  |
|  | **Current pregnancy** |  |  |
| FA58 | Number of antenatal care (ANC) | (numeric)  |__|__| |  |
| FA59 | Complication of current pregnancy  Several answers possible  - Preeclampsia with danger signs  - Preeclampsia without danger signs  - High blood pressure during pregnancy  - Eclampsia  - Placenta previa  - Intrauterine growth retardation (IUGR)  - Placental abruption  - Abnormalities of amniotic fluid (oligoamnios / hydramnios)  - Malaria  - Severe anemia  - Cholestasis of pregnancy  - Maternal Infection  - Premature rupture of membranes  - Chorio-amnionitis  - Preterm labor / preterm delivery  - Chronic Maternal Pathology  - Stopped Pregnancy  - Post – term pregnancy  - Other | (series of click)  |__|  |__|  |__|  |__|  |__|  |__|  |__|  |__|  |__|  |__|  |__|  |__|  |__|  |__|  |__|  |__|  |__|  |__|  |__| | If other, go to FA60 If no, go to FA61 |
| FA60 | If other complication, specify | (open field) |  |
| FA61 | Twin pregnancy? | no=0, yes=1 | If no, go to FA63 |
| FA62 | If yes, please specify the presentation of T1 | Cephalic = 1  Face = 2  Front = 3  Breech = 4  Transverse = 5  Other = 6 |  |
| FA63 | Triplet pregnancy or more? | no=0, yes=1 |  |
| FA64 | Intrauterine growth restriction? | no=0, yes=1 | If no, go to FA66 |
| FA65 | If yes, specify: | birth weight less than the 3rd percentile = 1    birth weight less than the 10th percentile = 2    Not documented = 9 |  |
| FA66 | Fetal or T1 weight (in g) last echography | not documented=99 99  (numeric)  |__|__|__|__|g |  |
| FA67 | T2 weight (in g) last echography | Not applicable = 8888 (if FA 61=no and FA 63=no)  not documented=99 99  (numeric)  |__|__|__|__|g |  |
| FA68 | T3 weight (in g) last echography | Not applicable = 8888 (if FA63=no)  not documented=99 99  (numeric)  |__|__|__|__|g |  |
| FA69 | Date last echography | not documented=09 09 9999  (dd/mm/yyyy) |  |
| FA70 | Duration of pregnancy (week) in the last echography | not documented = 99  (numeric)  |__|__| weeks |  |
|  | **Obstetrical examination before the decision of caesarean** |  |  |
| FA71 | Duration of pregnancy in weeks | not documented=99  (numeric)  |__|__| weeks | If the duration available on weekdays go to FA73 |
| FA72 | Duration of pregnancy in month | not documented=99  (numeric)  |__|__| months |  |
| FA73 | If less than 37 weeks has there been corticosteroid therapy? | Yes=1,  no = 0,  not documented= 9 |  |
| FA74 | Uterine height (cm)  (en cm) | (numeric)  |__|__| cm |  |
| FA75 | Fetal heart rate perceived? | Yes=1,  no = 0,  not documented= 9 |  |
| FA76 | Anomaly of fetal heart rate? | Yes=1,  no = 0,  not documented= 9 |  |
| FA77 | Cervical dilation (cm) | not documented=99  (numeric)  |__|__| cm |  |
| FA78 | Status of membranes | ruptured =1  intact=0 | if 0 go to FA82 |
| FA79 | If ruptured membranes specify date | not documented=09 09 9999  (dd/mm/aaaa) |  |
| FA80 | If ruptured membranes specify time | not documented=99 99  (hh:mm) |  |
| FA81 | If ruptured clarify the status of amniotic fluid: | Clear = 0  Tinted = 1  not documented= 9 |  |
| FA82 | Vaginal bleeding? | Yes=1  no = 0  not documented= 9 |  |
| FA83 | Presentation | Cephalic = 1  Face = 2  Front = 3  Breech = 4  Transverse = 5 |  |
| FA84 | Descent of the presentation : | Engaged =0  Not engaged =1  Not documented=9 |  |
| FA85 | Pelvis | Normal = 1  Asymmétric = 2  Narrow = 3  Not documented = 9 |  |
| FA86 | Systolic blood pressure (mm Hg) | not documented= 999  (numeric)  |__|__|__|mmHg |  |
| FA87 | Diastolic blood pressure (mm Hg) | not documented= 999  (numeric)  |__|__|__|mmHg |  |
| FA88 | Signs of moderate / severe preeclampsia? | Yes=1,  no = 0,  not documented= 9 |  |
| FA89 | Failed medical therapy (antihypertensive and anticonvulsant)? | Yes=1,  no = 0,  not documented= 9 |  |
| FA90 | Convulsions (Eclampsia)? | Yes=1,  no = 0,  not documented= 9 |  |
| FA91 | Signs of abruption placentae? | Yes=1,  no = 0,  not documented= 9 |  |
| FA92 | Maternal fever? | Yes=1,  no = 0,  not documented= 9 |  |
|  | **Labor monitoring** |  |  |
| FA93 | Was the woman in labor? | no=0, yes=1 | If no, go to  FA134 |
| FA94 | Was the partograph used? | no=0, yes=1 |  |
| FA95 | Stopping the dilation ? | Yes=1,  no = 0,  not documented= 9 | If no, go to FA97 |
| FA96 | If yes, duration of dilation stopping. | < 4hours = 1  From 4 to 6 hours= 2  > 6hours = 3  not documented = 9 |  |
| FA97 | Default of engagement at full dilation > 3 hours? | Yes=1,  no = 0,  not documented= 9 |  |
| FA98 | Induction of labor? | Yes=1,  no = 0,  not documented= 9 | If no, go to FA104 |
| FA99 | If yes, induction date | not documented=09 09 9999  (dd/mm/yyyy) |  |
| FA100 | If yes, induction time | not documented= 99 99  (hh:mm) |  |
| FA101 | Indication of tripping: multiple answers   - Maternal pathology - Fetal pathology - Post-term - Patient request - Other | (series of click)  *|__|*  *|__|*  *|__|*  *|__|*  *|__|* | If other, go to FA102 If no, go to FA103 |
| FA102 | If other specify | (open field) |  |
| FA103 | Failure of induction ? | Yes=1,  no = 0,  not documented= 9 |  |
| FA 104 | Active phase achieved? | no=0, yes=1 | If no go toFA108 |
| FA105 | Date start of active phase of labor | not documented=09 09 9999  (dd/mm/yyyy) |  |
| FA106 | Time start of active phase of labor | not documented= 99 99  (hh:mm) |  |
| FA107 | Duration of active phase of labor (hours) | not documented= 99  (numeric)  |__|__|hours |  |
| FA108 | Labour latency phase duration (in hours) | not documented= 99  (numeric)  |__|__|hours |  |
| FA109 | Signs of pre-rupture or uterine rupture? | Yes=1,  no = 0,  not documented= 9 |  |
| FA110 | A direction of laor has been attempted? | Yes=1,  no = 0,  not documented= 9 | If no, go to  FA120 |
| FA111 | Artificial rupture of membranes? | Yes=1,  no = 0,  not documented= 9  non applicable=8 (if FA78=1) | If no, go to FA120 |
| FA112 | If yes, date of artificial rupture of membranes | not documented=09 09 9999  (dd/mm/yyyy) |  |
| FA113 | If yes, time of artificial rupture of membranes | not documented= 99 99  (hh:mm) |  |
| FA114 | If yes, status of the amniotic fluid | Tinted = 1  Clear = 0  Not documented= 9 |  |
| FA115 | Delay realization caesarean after artificial rupture of membranes (RAM) | <4hours after ARM = 1  From 4 to 6 hours after ARM = 2  >6hours after ARM =3 | If answer = 1 go to FA120 |
| FA116 | Cervical dilation 4 hours after rupture of the membranes (in cm)? | not documented= 99  (numeric)  |__|__|cm |  |
| FA117 | Adequate uterine contractions (rhythm and intensity) 4 hours after rupture of membranes? | Yes=1,  no = 0,  not documented= 9 | If FA115=2 go to FA120 |
| FA118 | Cervical dilatation six hours after the rupture of membranes (in cm)? | not documented= 99,  (numeric)  |__|__| cm |  |
| FA119 | Were uterine contractions adequate (rhythm and intensity) 6 hours after rupture of membranes? | Yes=1,  no = 0,  not documented= 9 |  |
| FA120 | Anomaly of fetal heart rate during labor? | Yes=1,  no = 0,  not documented= 9 | If no go toFA126 |
| FA121 | If yes, start date of this anomaly | not documented=09 09 9999  (dd/mm/yyyy) |  |
| FA122 | If yes, start time of this anomaly | not documented= 99 99  (hh:mm) |  |
| FA123 | If yes, what was the treatment received? | Oxygenotherapy = 1  Lateral decubitus position = 2  Other measures = 6  No action = 3  Not documented = 9 | If other, go to FA124, If no go to FA125 |
| FA124 | If other treatment specify | (open field) |  |
| FA125 | Persistent anomaly after treatment? | Yes=1,  no = 0,  not documented= 9 |  |
| FA126 | Oxytocin? | Yes=1,  no = 0,  not documented= 9 | If no, go to  FA130 |
| FA127 | If yes, start date | not documented=09 09 9999  (dd/mm/yyyy) |  |
| FA128 | If yes, start time | not documented 99 99  (hh:mm) |  |
| FA129 | If yes, total dose infused before caesarean (in IU) | not documented 99 99  (numeric)  |__|__| IU |  |
| FA130 | Attempted instrumental delivery? | Yes=1,  no = 0,  not documented= 9 | If no, go to  FA134 |
| FA131 | If yes date of instrumental delivery attempt | not documented=09 09 9999  (dd/mm/yyyy) |  |
| FA132 | If yes attempt to instrumental delivery time | not documented 99 99  (hh:mm) |  |
| FA133 | If yes, technique employed | Forceps = 1  Vacuum = 2 |  |
|  | **Indication of caesarean section** |  |  |
| FA134 | Date indication caesarean | not documented=09 09 9999  (dd/mm/yyyy) |  |
| FA135 | Time indication caesarean | not documented 99 99  (hh:mm) |  |
| FA136 | Caesarean before labor? | Yes=1,  no = 0,  not documented= 9 |  |
|  | **Indication(s) of caesarean section** |  |  |
| FA137 | Fetal distress | no=0, yes=1 |  |
| FA138 | Long labor | no=0, yes=1 |  |
| FA139 | Preeclampsia | no=0, yes=1 |  |
| FA140 | History of caesarean | no=0, yes=1 |  |
| FA141 | If other indications, specify: multiple answers possible  - Placenta previa  - Placental abruption  - Abnormal or vicious presentation (front / transverse / hand / shoulder / posterior chin)  - Intrauterine growth restriction  - Post-term / term exceeded / prolonged pregnancy  - Vaginal bleeding late in pregnancy  - Multiple pregnancy / twin second retention  - Uterine Rupture  - Failed induction / caused dystocia  - Caesarean section for tubal sterilization  - Maternal request  - HIV  - Genital herpes / vulvar condylomas  - A history of rectal/ bladder / vaginal fistula  - pelvis suspect / pathological / unbalanced / limit / immature / narrow  - Precious pregnancy  - Prolapsed cord / funicular dystocia | (series of click)  *|__|*  *|__|*  *|__|*  *|__|*  *|__|*  *|__|*  *|__|*  *|__|*  *|__|*  *|__|*  *|__|*  *|__|*  *|__|*  *|__|*  *|__|*  *|__|*  *|__|* |  |
| FA142 | Signs of acute fetal distress at the moment of the indication of caesarean section? | Yes=1,  no = 0,  not documented= 9 |  |
| FA143 | Patient informed of the decision of caesarean? | Yes=1,  no = 0,  not documented= 9 |  |
| FA144 | Date incision | not documented=09 09 9999  (dd/mm/yyyy) |  |
| FA145 | Time incision | not documented 99 99  (hh:mm) |  |
| FA146 | Date extraction | not documented 09 09 9999  (dd/mm/yyyy) |  |
| FA147 | Time extraction | not documented 99 99  (hh:mm) |  |
| FA148 | Status newborn at birth or (T1) | Living =1  apparent death = 2  recent stillborn 3 =  macerated stillborn = 4  not documented = 9 |  |
| FA149 | Status T2 at birth | Living 1 =  apparent death = 2  recent stillborn 3 =  macerated stillborn = 4  not documented = 9  not applicable = 8 (if FA 61=no and FA 63=no) |  |
| FA150 | Status T3 at birth | Living =1  apparent death = 2  recent stillborn 3 =  macerated stillborn = 4  not documented = 9  not applicable = 8 (if FA63=no) |  |
| FA151 | Newborn or T1 resuscitate? | Yes=1,  no = 0,  not documented= 9 |  |
| FA152 | T2 resuscitate? | Yes=1,  no = 0,  not documented= 9  non applicable= 8 (if FA 61=no and FA 63=no) |  |
| FA153 | T3 resuscitate? | Yes=1,  no = 0,  not documented= 9  not applicable= 8(if FA63=no) |  |
| FA154 | If yes, duration of newborn or T1 resuscitation (in minutes) | not documented= 99  (numeric) |__|__| min |  |
| FA155 | If yes, duration of T2 resuscitation (in minutes) | not documented= 99  not applicable= 88 (if FA 61=no and FA 63=no)  (numeric) |__|__| min |  |
| FA156 | If yes, duration of T3 resuscitation (in minutes) | not documented= 99  not applicable= 88 (if FA63=no)  (numeric) |__|__| min |  |
| FA157 | Birth weight of newborn or T1 (in g) | not documented= 99 99  (numeric) |__|__|__|__| g |  |
| FA158 | Birth weight of T2 (en g) | not documented= 99 99  not applicable= 88 88 (if FA 61=no and FA 63=no)  (numeric) |__|__|__|__| g |  |
| FA159 | Birth weight of T3 (en g) | not documented= 99 99  not applicable= 88 88  (if FA63=no)  (numeric) |__|__|__|__| g |  |
| FA160 | Malformation ? | Yes=1,  no = 0,  not documented= 9 |  |
| FA161 | If yes, what type | (open field) |  |
|  | **Caesarean technique** |  |  |
| FA162 | Type of anesthesia | Spinal = 1 General = 2 Other = 6 |  |
| FA163 | If other, specify | (open field) |  |
| FA164 | Antibiotic prophylaxis? | Yes=1,  no = 0,  not documented= 9 | If no, go to  FA168 |
| FA165 | If yes, date of administration | not documented 09 09 9999  (dd/mm/yyyy) |  |
| FA166 | If yes, time of administration | not documented 99 99  (hh:mm) |  |
| FA167 | If yes, specify antibiotic used | Ampicillin=1  Amoxicillin=2  Amoxicillin+ clavulanic acid =3  Ceftriaxon=4  Other=6 | If other, go to FA168 If no, go to FA169 |
| FA168 | If other, specify | (open field) |  |
| FA169 | If yes, specify dose (in g) | (numeric) |__|g |  |
| FA170 | Type of cutaneous incision | Joel Cohen = 1  Median = 2  other = 6 |  |
| FA171 | If other specify | (open field) |  |
| FA172 | Type of uterine incision | Segmental = 1  Corporeal or segmento-corporeal = 2  Other = 6 | If other, go to FA173 If no, go to FA174 |
| FA173 | If other specify | (open field) |  |
| FA174 | Placental delivery mode | By controlled traction = 1 Manual = 2  Not documented = 9 |  |
| FA175 | Technique of parietal closure | According to Starck (closed in two layers) = 1  Layer by layer = 2  Other = 6 | If other, go to FA176 If no, go to FA177 |
| FA176 | If another technique, specify | (open field) |  |
|  | **Monitoring after caesarean** |  |  |
| FA177 | Regular blood pressure measurement two hours after caesarean section? | Yes=1,  no = 0,  not documented= 9 |  |
| FA178 | Diuresis measured after caesarean section? | Yes=1,  no = 0,  not documented= 9 |  |
| FA179 | Bleeding monitoring? | Yes=1,  no = 0,  not documented= 9 |  |
| FA180 | Patient eligible for anti thromboembolic prophylaxis? | Yes=1,  no = 0,  not documented= 9 | If no, go to  FA186 |
| FA181 | Anti thromboembolic prophylaxis conducted? | Yes=1,  no = 0,  not documented= 9 | If no, go to  FA186 |
| FA182 | If yes, start date of anti thromboembolic prophylaxis | not documented 09 09 9999  (dd/mm/yyyy) |  |
| FA183 | If yes, start time of anti thromboembolic prophylaxis | not documented 99 99  (hh:mm) |  |
| FA184 | If yes, what was the molecule used | Enoxaparin sodium = 1  Fraxiparin = 2  Calciparin = 3  Others = 6 |  |
| FA185 | If other specify molecule used | (open field) |  |
| FA186 | Liquid diet six hours after caesarean? | Yes =1,  no = 0,  not documented= 9 |  |
| FA187 | Discharge / death / transfer date | not documented 09 09 9999  (dd/mm/yyyy) |  |
| FA188 | Discharge / death / transfer time | not documented 99 99  (hh:mm) |  |
| FA189 | Status of the patient at discharge | alive =1  dead=0 |  |
| FA190 | Newborn or T1 status at discharge | alive = 1  dead before 24 hours = 2  dead after 24 hours = 3 |  |
| FA191 | T2 status at discharge | alive = 1  dead before 24 hours = 2  dead after 24 hours = 3  Not applicable = 8 (if FA 61=no and FA 63=no) |  |
| FA192 | T3 status at discharge | alive = 1  dead before 24 hours = 2  dead after 24 hours = 3  Not applicable = 8 (if FA63=no) |  |

# Appendix 2: Criteria for non-medically indicated caesareans

| Previous caesarean section  Pre-labour caesarean not indicated if*:   - One previous caesarean section with transversal scar - Singleton foetus in cephalic presentation - Lack of ultrasound or clinical evidence of macrosomia - Lack of radiographic or clinical evidence of restricted pelvis - Possibility of performing emergency intra-partum caesarean section 24h on call   Caesarean during labour not indicated if*:   - Same criteria as pre-labour caesarean (see above) - Cervical dilation progressing normally (>2cm in 4 h) - Presentation progressing normally at full dilation (no arrest >3h) - Lack of evidence of foetal distress** - No documented signs of uterine rupture | Prolonged/obstructed labour  Pre-labour caesarean for presumed obstructed labour not indicated if*:   - Singleton foetus in cephalic presentation - Lack of ultrasound or clinical evidence for macrosomia - Lack of radiographic or clinical evidence of restricted pelvis - No documented history of fistula or uterine malformation - No documented history of uterine rupture   Caesarean for failed induction or slow dilation without foetal distress is not indicated if*:   - Membranes intact   OR   - Membranes ruptured, uterine contractions adequate and time elapsed since stagnation of dilation under 4h   OR   - Membranes ruptured, uterine contractions inadequate and time elapsed since stagnation of dilation under 6h   Caesarean for no descent of presentation without foetal distress not indicated if*:   - Time elapsed between full dilation and caesarean under 3h - Instrumental delivery (forceps or vacuum) not attemped |
| --- | --- |
| Pre-eclampsia  Pre-labour caesarean not indicated if*:   - Lack of evidence of foetal distress** - Lack of clinical or ultrasound evidence of foetal growth restriction - No documented signs of severity for the woman° - No documented signs of abruptio placentae   Caesarean during labour not indicated if*:   - Same criteria as pre-labour caesarean (see above) - Cervical dilation progressing normally (>2cm in 4h)   Presentation progressing normally at full dilation (no arrest >3h) | Foetal distress  Caesarean during labour not indicated if*:signs of abruptio placentae   - Clear amniotic fluid or not documented - No maternal fever or not documented - Cervix dilation or presentation progressing normally - Foetal heart rate normal (120–160 beats/min.) OR abnormal but uncorrected (oxygen administration and mother on left side) |

* All criteria are required.

** Signs of foetal distress: foetal heart rate abnormalities (<120 or >160 beats/min. or repeated decelerations); coloured amniotic fluid.

° Signs of severe pre-eclampsia: blood pressure ≥160/110 mmHg; albuminuria ≥3+ or ≥3 g/24h; oliguria <30 mL/h; headache; epigastric pain; vision disorders; neurologic disorders; seizures; hemolysis; low platelet count; high liver enzymes.

# Appendix 3: Analysis grid for caesarean sections audit

1. **The aims**

The "analysis grid" is a tool for evaluators (audit committee) to:

- Submit a repertoire of key questions to identify cases of non medically justified caesareans.
- Differentiate medical and non-medical aspects (related to the structure) referring to the management of labor and the decision of caesarean sections.
- Identify the elements that need to be strengthened or changed in current practice to reduce the number of not medically justified caesarean.

1. **The conduct of the caesareans audit session.**

The review of practice in each case include three distinct phases:

1. Chronological reconstruction of management of the patient who underwent caesarean section since her arrival to the hospital for delivery until her discharge; with the identification of positive and negative events in the process of this medical management (management of labor, rationality of indications of caesarean sections)..
2. Identification of factors that may have prevented the adequate care of the patient and the appropriate decision for caesarean section.
3. Propose solutions to ensure the best possible care for future deliveries.
4. **CHRONOLOGY OF THE MANAGEMENT OF THE PATIENT**

The audit starts with the reconstruction of chronology of the management elements of each patient upon her arrival at the hospital until her discharge. The audit team will highlight the strengths and weaknesses of the management process in the hospital. The strengths and weaknesses will be different from one patient to another.

.

The main key events to consider are listed below.

**A.1 Admission**

- The personal information concerning the patient have they been indicated? (name, surname, age, address))
- Was the patient referred? If so, from where was she evacuated? what was the reference pattern?
- What was the reason for admission of the patient?
- Were the medical history of the patient recorded?
- Were the obstretical history of the patient recorded ?
- Were the surgical history of the patient recorded ?
- Were the complications of current pregnancy indicated if any??
- Was the patient monitored prenatally? If so, was the patient considered at risk of complication for this pregnancy? was the patient benefit from elective caesarean section before labor?

**A.2 Obstetrical examination and diagnosis**

- The initial obstetrical examination was done adequately technically ?
- Was the initial diagnosis proper?
- Was all the necessary investigations made?
- All the investigations made were they necessary?
- The results have they been used ?

**A.3 Labor monitoring**

- The monitoring of labor has been well documented? This may include notification of diagnosis hours, of complications management.
- Was the labor monitoring adequate? For example, this may include the use of the partograph, diagnosis and appropriate management of complications arising during delivery (dystocia, preeclampsia / eclampsia, fetal distress etc.)

**A.4 Indication of caesarean section**

- The indication for caesarean has it been well documented?
- The indication was it adequate?

**A.7 Record Keeping**

- The information contained in the medical chart was complete?
- If not, make a list specifying the missing information
- The information contained in the medical chart was it appropriate for: assess the monitoring of labor, the rationality of the indication and the quality of caesarean section and postoperative monitoring?
- If not, make a list specifying the information that should have been included and which have not been.

1. **FACTORS THAT EXPLAIN THE POSITIVE AND NEGATIVE EVENT IN THE MANAGEMENT OF THE PATIENT**

- Is it a problem?

- On admission

- During labor monitoring

- When indicated caesarean

- Upon achievement of the caesarean

- During monitoring

- Is it a problem?

- The qualification of personnel

- Of equipment

- Availability of equipment

- Availability of drugs

- On the organization of care

- On affordability

1. **IDENTIFICATION OF FACTORS THAT CAN BE IMPROVED AND RECOMMENDATIONS**

- The final step in the audit process is to propose solutions to identified problems.
- The solutions will be specific to each hospital.
- The audit should focus on both positive and negative.
- The focus must be on the organizational aspects.

**C.1 The staff**

- Qualifications: was the person who performed a specific act qualified for this?
- Technical ability/ skill: the qualified person has sufficient competence or technical ability to carry out certain tasks?
- Always availability:

- The hospital has an anesthesiologist (doctor or nurse in anesthesia)?

- The hospital has an operator (doctor or nurse in surgery)?

- The hospital has a full time laboratory technician takes custody on site etc?

- Temporary availability:

- The hospital Has an anesthetist but he / she is on leave or fails to take custody there?

- The hospital has an operator but he / she is on leave or fails to take custody there?

- The hospital has a laboratory technician but he / she is on leave or fails to take custody there?

- Rotary :

- Has the key personnel taked custody?

- Has there been a staff member designated to alert custodial staff?

- Staff residence:

- On-duty staff live close enough to the hospital to be able to come on time?

- Location on-duty staff:

- Can it be localized in time?

- Did it adhere to the hospital guidelines about the availability during the guard?

- Supervision of junior staff: Supervision is it regular, effective and adequate for custody also (relative to the treatment, the diagnosis, the use of protocols)?
- Communication and relationship (between the medical staff and between staff and patient).

Note that these factors apply to all hospital staff who is involved in the care chain, regardless of its position. For example, a major delay to conduct certain key activities may be due to a combination of smaller time frames at different stages of care. It is important to note for every problem (competence to handle some complications, response time, etc.) what type of staff it?

**C.2 Equipement**

- Availability: equipment is it available permanently or intermittently in time, in space, in relation to needs? For example:

- Is the partograph available in the delivery room?

- The tools to monitor labor (Pinard stethoscope, ribbon, sphygmomanometer, gloves, clock etc.) are available in the delivery room?

- A forceps or vacuum was in the delivery room available and ready for use?

- Were the instruments of surgery and anesthesia available and ready for use?

- The operating kits and laboratory tools were they available?

- The supplies have been made on time?

- Functional: undamaged or broken

**C.3 Emergency drugs (eg oxytocin)**

- Always available
  - At the hospital pharmacy
  - At the emergency room
  - In the delivery room
  - In the operating room
- Intermittent availability
  - At the hospital pharmacy
  - At the emergency room
  - In the delivery room
  - In the operating room
- Accessibility
  - Locked
  - The staff of pharmacy can provide the medicines at any time
- Affordability: the patient provides medicines on time
  - At the emergency room
  - In the delivery room
  - In the operating room

**C.4 The infrastructure**

- Permanent or intermittent availability for example of the operating room, sterile surgical instruments, surgical equipment are the limited?

**C.5 Clinical decision rules**

- Are there standardized protocols?
- Are there any algorithms (clinical decision tree)?
- Are they available in the delivery room?

**C.6 Organization and Management**

- Arrangements are being taken to ensure the availability of staff?
- Arrangements are being taken to facilitate access to the operating room?
- The directives and regulations are they contrary to the quality of services?
- What is the relationship between medical staff and administrative staff?
- What is the quality of communication between the various units of the service and within each unit?
- The consumables are they renewed?

**C.7 Patient and family**

- Ability to pay; specify that the patient was able to pay or can not pay
- Cooperation of the family

**C.8 Other (specify)**

**C.9 PROPOSE SOLUTIONS …**

# Appendix 4: Report of the audit session

Identification number of the health facility: /_/_/_/_/

Date of the audit session (dd/mm/yy) :  /  /

Number of persons present during the session:

Staff Category (number): Doctors  Midwifes  Nurses

Students  Administrative staff  Others staff: specify ___________________

Audit report completed by: ______________________________

Professional title _______________

Checking the implementation of the recommendations of the subsequent meeting: Done □ NA □ (if first session)

Did all recommendations have been implemented? Yes □ No□ NA (if first session) □

If no, why?

____________________________________________________________________________________________________________________________________________________________________________________________________________________________________________________________________________________________________________________________________________________

Please indicate what measures have not been implemented:

____________________________________________________________________________________________________________________________________________________________________________________________________________________________________________________________________________________________________________________________________________________

What are the decisions to be adopted to ensure that measures will be implemented?

____________________________________________________________________________________________________________________________________________________________________________________________________________________________________________________________________________________________________________________________________________________

**Rationality of diagnosis for cases audited during this session**

| **Indication** | **Total number of caesareans** | **Number of non-medically indicated caesareans** | **% of non-medically indicated caesareans** |
| --- | --- | --- | --- |
| Prolonged / obstructed labor |  |  |  |
| Pre-eclampsia/eclampsia |  |  |  |
| Previous caeserean section |  |  |  |
| Fœtal distress |  |  |  |
| **Total** |  |  |  |

Main problems identified in the management of cases audited?

____________________________________________________________________________________________________________________________________________________________________________________________________________________________________________________________________________________________________________________________________________________

__________________________________________________________________________________________________________________________________________________________________________

What are the positive points in the management of cases audited?

____________________________________________________________________________________________________________________________________________________________________________________________________________________________________________________________________________________________________________________________________________________

What are the main causes of malfunctions identified?

- Staff (qualification, skills, availability, attitude, communication)

- Drugs (availability, accessibility)

- Equipment (availability, accessibility, functional)

- Protocols (existence, availability, appropriation, use)

- Management, organization of care

- Patient and family (access to care, responsibility, and beliefs)

**Measures** to be implemented (include solutions to achieve the measures of the latter (s) session (s) that have not yet been implemented:

|  | **Measures** | **Person responsible for implementation** | **Date of implementation** | **Person responsible for confirming that the measures were implemented** |
| --- | --- | --- | --- | --- |
| 1. |  |  |  |  |
| 2. |  |  |  |  |
| 3. |  |  |  |  |
| 4. |  |  |  |  |
| 5. |  |  |  |  |
| 6. |  |  |  |  |
| 7. |  |  |  |  |
| 8. |  |  |  |  |
| 9. |  |  |  |  |
| 10. |  |  |  |  |
