# Supplementary material for: DECIDE: a cluster randomized controlled trial to reduce non-medically indicated caesareans in Burkina Faso
Source: BMC Pregnancy Childbirth. 2016 Oct 21;16:322. doi: 10.1186/s12884-016-1112-8 (PMC5073955; doi:10.1186/s12884-016-1112-8)
Supplement: Additional file 9: — Qualitative study tools. (DOC 51 kb) [file 12884_2016_1112_MOESM9_ESM.doc]

**PERCEPTION ON THE RELEVANCE AND EFFECTIVENESS TRAINING WORKSHOP OF MATERNITY SERVICES MANAGERS ON CAESAREAN PRACTICE**

**INTERVIEW GUIDE**

**Introduction**

*Introduce themselves and present the objectives of qualitative research (analyze the implementation process of the project, its sustainability and its effects)
Aspects relating to confidentiality and consent*

**Presentation**

- Can you briefly introduce and present your service?

**Perceptions regarding the problematic of non-medically indicated caesarean section (NMICS)**

In light of yesterday's discussion on **NMICS**: Do you think that in your department there is a relatively high number of NMJCS?

If yes:

- Is the problem rather concern in your service or minor? Why ?
- How can one explain the number of NMICS that you have in your service?
- How do you stand with the services here (workshop) represented?
- Can you tell us more about the influence of certain political measures such as subvension of EmONC on the high number of NMICS?
- Is there's anything else to say on NMICS in your service?

**Relevance of the workshop**

- What do you think of the usefulness of this workshop?
- How does the workshop meet your needs?
- How the workshop does not meet your needs?
- To what extent the participation of the workshop will improve the practice of cesarean section in your department? (be specific)
- How well it will not improve the practice of cesarean section in your department?
- What the general interaction do you think (between participants /between participants and trainers)?
- How do you see the next of the intervention?
- How do you see the after-intervention?
- Other comments about the workshop?

**QUALITATIVE INTERVIEW ON THE IMPLEMENTATION**

**INTERVIEW GUIDE**

**Introduction**

*Introduce themselves and present the objectives of qualitative research (analyze the implementation process of the project, its sustainability and its effects)*

*Aspects relating to confidentiality and consent*

**Presentation**

• Can you briefly introduce and present your service?

**Analysis of the problematic OF non-medically** INDICATED caesarean **section (NMICs)**

**PROBLEMATIC OF NMJC IN THE HEALTH FACILITY**

- What are your appreciations regarding the rate of NMIC in your health facility?
- How do you explain this?
- What are the social contexts that explain this rate?
- Did the subvension of EmONC influence the practice of cesarean? How and why?
- Did the implementation of performance-based funding influence the practice of cesarean? How and why?
- What are the mechanisms and actions that can help reduce the rate of NMICS (at the actor-providers)

**Analysis of the intervention**

**RESTITUTION OF TRAINING (ADDRESSED TO THE HEAD OF DEPARTMENT)**

• How many restitution trainings were conducted in your department? Why?

•Who was the target audience?

•How did participants selected?

• What were the main challenges associated with the preparation and implementation of these restitution?

• What are the themes that have interested most participants?

• What are the themes that have interested the least participants?

• What do you think of the effectiveness of these refunds?

• What could be done to improve these restitutions?

• How the proposed algorithms are useful to you?

• How do you consult themes?

**clinical Audit**

• How the committee responsible for the organization of clinical audits has been implemented?

•What do you think of this meeting clinical audit of caesarean sections?

•What were the difficulties you encounter before clinical audit?

•What were the origins of these problems?

•Does Clinical audit has met your needs?

•In your opinion, what is the usefulness of this strategy?

•In what ways clinical audit can improve your practice?

•In your opinion, how clinical audit can only improve your practice?

• How do these audits can be improved?

**Decision-support reminders**

• How many messages did you receive?

• Do you know who sends you?

• In your opinion, what is the purpose of these messages?

• What do you think of this strategy?

• Are these messages understanding?

• Are these messages helpful for you?

• All your information needs and technical support are they covered?

• What are the non-covered needs?

• What can be improved in the implementation of this strategy?
