# Supplementary material for: DECIDE: a cluster randomized controlled trial to reduce non-medically indicated caesareans in Burkina Faso
Source: BMC Pregnancy Childbirth. 2016 Oct 21;16:322. doi: 10.1186/s12884-016-1112-8 (PMC5073955; doi:10.1186/s12884-016-1112-8)
Supplement: Additional file 10: — List of experts. (DOC 26 kb) [file 12884_2016_1112_MOESM10_ESM.doc]

**List of experts**

|  |
| --- |

- Nils Chaillet : University of Sherbrooke

- Patrick Rozenberg : CHU POISSY Paris, France

- Cyrille Huchon : CHU POISSY Paris, France

- Arnaud Fauconnier : CHU POISSY Paris, France

- Seni Kouanda : IRSS Ouagadougou, Burkina Faso

- Benjamin Hounkpatin : CHU HOMEL Cotonou, Bénin

- René Perrin : CHU HOMEL Cotonou, Bénin

- Mamadou Traoré : URFOSAME BAMAKO, Mali

- Marie Hélène Bouvier Colle : Hôpital Port Royal Paris, France

- Catherine Deneux-Tharaux : Hôpital Port Royal Paris, France

- Charlemagne Ouédraogo : CHU Yalgado Ouédraogo Ouagadougou, Burkina Faso

- Fabienne Richard: Institute of Tropical Medicine

- Luc de Bernis : UNFPA

- Anne Rousseau : CHU POISSY Paris, France

- Ali Ouédraogo : CHU Yalgado Ouédraogo Ouagadougou, BURKINA FASO

-Emmanuel Bujold : University of Laval, Canada
