# Supplementary material for: DECIDE: a cluster randomized controlled trial to reduce non-medically indicated caesareans in Burkina Faso
Source: BMC Pregnancy Childbirth. 2016 Oct 21;16:322. doi: 10.1186/s12884-016-1112-8 (PMC5073955; doi:10.1186/s12884-016-1112-8)
Supplement: Additional file 11: — Caesarean section form. (DOC 285 kb) [file 12884_2016_1112_MOESM11_ESM.doc]

# caesarean section form

| **Variable** | **Question** | **Code** | **Jump** |
| --- | --- | --- | --- |
| FA01 | Hospital name | RH Dédougou = 001  MCS Solenzo = 002  RH Banfora = 003  MCS Bogodogo = 004  MCS Boulmiougou = 005  MCS Nongr-Massom= 006  RH Tenkodogo = 007  MCS Koupéla = 008  RH Kaya = 009  MCS Boulsa = 010  RH Koudougou = 011  RH Fada = 012  MCS Do = 013  MCS Dafra = 014  MCS Houndé = 015  MCS Orodara = 016  RH Ouahigouya = 017  MCS Yako = 018  MCS Zorgho = 019  RH Dori = 020  MCS Djibo = 021  RH Gaoua = 022 |  |
| FA02 | Patient name | (Open field) |  |
| FA03 | Patient surname | (Open field) |  |
| FA04 | Patient age (years) | (numeric) |__|__|years |  |
| FA05 | Lieu de résidence de la patiente | Rural = 1  Urban parceled = 2  Urban non parceled = 3 |  |
| FA06 | Name district / area / village patient | (Open field) |  |
| FA07 | Patient phone | (numeric) |  |
| FA08 | Patient weight beginning pregnancy | (numeric)|__|__|__|Kg |  |
| FA09 | Patient weight ending pregnancy | (numeric)|__|__|__|Kg |  |
| FA10 | Name provider indicated caesarean section | (a list will be proposed for each hospital after the inclusion visit)  |__|__|__| see codes |  |
| FA11 | Surname provider indicated caesarean section | (a list will be proposed for each hospital after the inclusion visit) |  |
| FA12 | Name provider has realized the caesarean | (a list will be proposed for each hospital after the inclusion visit)  |__|__|__| see codes |  |
| FA13 | Surame provider who realized the caesarean | (a list will be proposed for each hospital after the inclusion visit) |  |
| FA14 | Date admission | (dd/mm/yyyy) |  |
| FA15 | Time admission | (hh:mm) |  |
| FA16 | Woman admitted after a reference? | no=0, yes=1 | If no go to FA22 |
| FA17 | Type health facility who referred the woman | Primary health center = 1  District hospital = 2  Regional hospital = 3  Other = 6 |  |
| FA18 | Name health facility who referred the woman | (a list will be proposed for each hospital after the inclusion visit) |  |
| FA19 | Distance (km) between hospital who made the referral and this hospital | (numeric)|__|__|__| Km |  |
| FA20 | Reason (s) Reference  Many possible responses  - Labour on term pregnancy  - Labour on scarred uterus  - engagement failure at full dilation  - Failure of induction  - Retention of the second twin  - Vicious Presentation  - Abnormalities of amniotic fluid  - Precious Pregnancy  - Premature rupture of membranes  - Premature delivery threat  - Hypertension in pregnancy  - Eclampsia / HELLP syndrome  - Maternal Pathology on pregnancy  - Anaemia in pregnancy  - Pregnancy endded / not active or intrauterine fetal death (IUFD) or retention of death egg (ROM)  - Post-term pregnancy  - Malaria on pregnancy  - Cephalopelvic disproportion  - Fetal distress  - Pathological / limit / unbalanced / narrowed / immature pelvis  - Placenta previa  - Fetal macrosomia  - Pre-rupture / uterine rupture  - Vaginal bleeding at the end of pregnancy  - Urinary tract infection during pregnancy  - Placental abruption  - Vulvar / perineal condylomata  - Multiple pregnancy  - HIV on pregnancy  - Cord Abnormalities (prolapsed cord / cord circular)  - Other | (series of click)  |__|  |__|  |__|  |__|  |__|  |__|  |__|  |__|  |__|  |__|  |__|  |__|  |__|  |__|  |__|  |__|  |__|  |__|  |__|  |__|  |__|  |__|  |__|  |__|  |__|  |__|  |__|  |__|  |__|  |__|  |__| |  |
| FA21 | If other, specify | (Open field) |  |
| FA22 | Reason for admission  Many possible responses   - - Labour on term pregnancy - - Labour on scarred uterus - - engagement failure at full dilation - - Failure of induction - - Retention of the second twin - - Vicious Presentation - - Abnormalities of amniotic fluid - - Precious Pregnancy - - Premature rupture of membranes - - Premature delivery threat - - Hypertension in pregnancy - - Eclampsia / HELLP syndrome - - Maternal Pathology on pregnancy - - Anaemia in pregnancy - - Pregnancy endded / not active or intrauterine fetal death (IUFD) or retention of death egg (ROM) - - Post-term pregnancy - - Malaria on pregnancy - - Cephalopelvic disproportion - - Fetal distress - - Pathological / limit / unbalanced / narrowed / immature pelvis - - Placenta previa - - Fetal macrosomia - - Pre-rupture / uterine rupture - - Vaginal bleeding at the end of pregnancy - - Urinary tract infection during pregnancy - - Placental abruption - - Vulvar / perineal condylomata - - Multiple pregnancy - - HIV on pregnancy - - Cord Abnormalities (prolapsed cord / cord circular) - - Other | (series of click)  |__|  |__|  |__|  |__|  |__|  |__|  |__|  |__|  |__|  |__|  |__|  |__|  |__|  |__|  |__|  |__|  |__|  |__|  |__|  |__|  |__|  |__|  |__|  |__|  |__|  |__|  |__|  |__|  |__|  |__|  |__| |  |
| FA23 | If other, specify | (Open field) |  |
| **Socio-economic characteristics** | | | |
| FA24 | Patient education level | Unschooled illiterate = 0  Literate but not schooled = 1  Primary level = 2  Lower secondary = 3  Upper secondary school = 4  Higher educational level = 5 |  |
| FA25 | Spouse education Level | Unschooled illiterate = 0  Literate but not schooled = 1  Primary level = 2  Lower secondary = 3  Upper secondary school = 4  Higher educational level = 5 |  |
| FA26 | Patient profession | Housewife 1 =  Breeder / farmer = 2  Trader = 3  Employee = 4  Private employee = 5  Other = 6 | If other, go toFA27 If no go to FA28 |
| FA27 | If other, specify | (Open field) |  |
| FA28 | Spouse profession | Breeder / farmer =1  Trader = 2  Employee = 3  Private employee = 4    Other = 6 | If other, go toFA29 If no, go to FA30 |
| FA29 | If other, specify | (Open field) |  |
| FA30 | Total amount paid for drugs, consumables and other products paid in the HF where was held caesarean (CFA) | (numeric)  |__|__|__|__|__|CFA |  |
| FA31 | Total amount paid for acts of care in the HF where was held caesarean (CFA) | (numeric)  |__|__|__|__|__| CFA |  |
| FA32 | Total amount paid for transportation between the HF and who referred the hospital where they underwent caesarean delivery (CFA)? | not applicable= 88 888 (if FA16 = no)  |__|__|__|__|__| CFA |  |
| FA33 | Amount for hospitalization (CFA) | |__|__|__|__|__| CFA |  |
| **Possession of household property** | | | |
| FA34 | Does the household own a motorcycle? | no=0, yes=1 |  |
| FA35 | The household does have a car? | no=0, yes=1 |  |
| FA36 | Does the household own a fixed telephone? | no=0, yes=1 |  |
| FA37 | How many mobile phones does the household have?  (If no mobile phones put 0) | (numeric) |__| |  |
| FA38 | How many hens / Guinea fowl / turkey / duck does the household has?  (If no animals put 0) | (numeric)  |__|__|__|__| |  |
| FA39 | How many sheep / goats / pigs does the household have?  (If no animals put 0) | (numeric)  |__|__|__|__| |  |
| FA40 | How many cattle / camel / donkey does the household have?  (If no animals put 0) | (numeric)  |__|__|__|__| |  |
| FA41 | Does the household have drinking water? | no=0, yes=1 |  |
| FA42 | Type of household housing | Homeowner = 1  Rental housing = 2 |  |
| FA43 | Type the household dwelling | Made of cement = 1  Mudbrick = 2  Mixed = 3 |  |
| FA44 | Does the household have air conditioner ? | no=0, yes=1 |  |
| **Clinical history** | | | |
| FA45 | Medical  Many possible responses     - HIV - Diabetes - High blood pressure - Sickle Cell Disease - Asthma - Peptic ulcer - Heart Disease - Hepatitis - Tuberculosis - Toxoplasmosis - Rubella - Genital infection - Syphilis - Other diseases | (series of click)  *|__|*  *|__|*  *|__|*  |__|  *|__|*  *|__|*  *|__|*  *|__|*  *|__|*  *|__|*  *|__|*  *|__|*  *|__|*  *|__|* | If other, go toFA46 If no, go to FA47 |
| FA46 | If other pathology, specify | (Open field) |  |
|  | **Obstetric** |  |  |
| FA47 | Number of pregnancies | (numeric)  |__|__| | If 01 go to FA 56 |
| FA48 | Number of deliveries | (numeric)  |__|__| |  |
| FA49 | Number of previous abortions | (numeric)  |__|__| |  |
| FA50 | Number of previous caesarean | (numeric)  |__|__| | If 00 go to FA53 |
| FA51 | History of corporeal caesarean section? | Yes=1,  no = 0  not documented = 9 |  |
| FA52 | Date last caesarean | (dd/mm/yyyy)  not documented=09 09 9999 |  |
| FA53 | Date last birth | (dd/mm/yyyy)  not documented=09 09 9999 |  |
| FA54 | Other obstetric history | no=0, yes=1 | If no, go to  FA56 |
| FA55 | If other obstetric history, specify: | (Open field) |  |
|  | **Surgical** |  |  |
| FA56 | Surgical history of the patient  Many possible responses  - Polymyomectomie  - Cure of uterine malformation  - Cure of obstetric fistula  - Appendectomy  - Salpingectomy  - oophorectomy  - Peritonitis  - Bowel obstruction  - Conization / cryotherapy  - Cure of synechia  - Pelvic surgery  - Pelvic fracture  - Other | (series of click)  *|__|*  *|__|*  *|__|*  *|__|*  *|__|*  *|__|*  *|__|*  *|__|*  *|__|*  *|__|*  *|__|*  *|__|*  *|__|* | If other, go to FA57 If no, go to FA58 |
| FA57 | If other surgical history, specify | (Open field) |  |
|  | **Current pregnancy** |  |  |
| FA58 | Number of antenatal care (ANC) | (numeric)  |__|__| |  |
| FA59 | Complication of current pregnancy  Several answers possible  - Preeclampsia with danger signs  - Preeclampsia without danger signs  - High blood pressure during pregnancy  - Eclampsia  - Placenta previa  - Intrauterine growth retardation (IUGR)  - Placental abruption  - Abnormalities of amniotic fluid (oligoamnios / hydramnios)  - Malaria  - Severe anemia  - Cholestasis of pregnancy  - Maternal Infection  - Premature rupture of membranes  - Chorio-amnionitis  - Preterm labor / preterm delivery  - Chronic Maternal Pathology  - Stopped Pregnancy  - Post – term pregnancy  - Other | (series of click)  |__|  |__|  |__|  |__|  |__|  |__|  |__|  |__|  |__|  |__|  |__|  |__|  |__|  |__|  |__|  |__|  |__|  |__|  |__| | If other, go to FA60 If no, go to FA61 |
| FA60 | If other complication, specify | (open field) |  |
| FA61 | Twin pregnancy? | no=0, yes=1 | If no, go to FA63 |
| FA62 | If yes, please specify the presentation of T1 | Cephalic = 1  Face = 2  Front = 3  Breech = 4  Transverse = 5  Other = 6 |  |
| FA63 | Triplet pregnancy or more? | no=0, yes=1 |  |
| FA64 | Intrauterine growth restriction? | no=0, yes=1 | If no, go to FA66 |
| FA65 | If yes, specify: | birth weight less than the 3rd percentile = 1    birth weight less than the 10th percentile = 2    Not documented = 9 |  |
| FA66 | Fetal or T1 weight (in g) last echography | not documented=99 99  (numeric)  |__|__|__|__|g |  |
| FA67 | T2 weight (in g) last echography | Not applicable = 8888 (if FA 61=no and FA 63=no)  not documented=99 99  (numeric)  |__|__|__|__|g |  |
| FA68 | T3 weight (in g) last echography | Not applicable = 8888 (if FA63=no)  not documented=99 99  (numeric)  |__|__|__|__|g |  |
| FA69 | Date last echography | not documented=09 09 9999  (dd/mm/yyyy) |  |
| FA70 | Duration of pregnancy (week) in the last echography | not documented = 99  (numeric)  |__|__| weeks |  |
|  | **Obstetrical examination before the decision of caesarean** |  |  |
| FA71 | Duration of pregnancy in weeks | not documented=99  (numeric)  |__|__| weeks | If the duration available on weekdays go to FA73 |
| FA72 | Duration of pregnancy in month | not documented=99  (numeric)  |__|__| months |  |
| FA73 | If less than 37 weeks has there been corticosteroid therapy? | Yes=1,  no = 0,  not documented= 9 |  |
| FA74 | Uterine height (cm)  (en cm) | (numeric)  |__|__| cm |  |
| FA75 | Fetal heart rate perceived? | Yes=1,  no = 0,  not documented= 9 |  |
| FA76 | Anomaly of fetal heart rate? | Yes=1,  no = 0,  not documented= 9 |  |
| FA77 | Cervical dilation (cm) | not documented=99  (numeric)  |__|__| cm |  |
| FA78 | Status of membranes | ruptured =1  intact=0 | if 0 go to FA82 |
| FA79 | If ruptured membranes specify date | not documented=09 09 9999  (dd/mm/aaaa) |  |
| FA80 | If ruptured membranes specify time | not documented=99 99  (hh:mm) |  |
| FA81 | If ruptured clarify the status of amniotic fluid: | Clear = 0  Tinted = 1  not documented= 9 |  |
| FA82 | Vaginal bleeding? | Yes=1  no = 0  not documented= 9 |  |
| FA83 | Presentation | Cephalic = 1  Face = 2  Front = 3  Breech = 4  Transverse = 5 |  |
| FA84 | Descent of the presentation : | Engaged =0  Not engaged =1  Not documented=9 |  |
| FA85 | Pelvis | Normal = 1  Asymmétric = 2  Narrow = 3  Not documented = 9 |  |
| FA86 | Systolic blood pressure (mm Hg) | not documented= 999  (numeric)  |__|__|__|mmHg |  |
| FA87 | Diastolic blood pressure (mm Hg) | not documented= 999  (numeric)  |__|__|__|mmHg |  |
| FA88 | Signs of moderate / severe preeclampsia? | Yes=1,  no = 0,  not documented= 9 |  |
| FA89 | Failed medical therapy (antihypertensive and anticonvulsant)? | Yes=1,  no = 0,  not documented= 9 |  |
| FA90 | Convulsions (Eclampsia)? | Yes=1,  no = 0,  not documented= 9 |  |
| FA91 | Signs of abruption placentae? | Yes=1,  no = 0,  not documented= 9 |  |
| FA92 | Maternal fever? | Yes=1,  no = 0,  not documented= 9 |  |
|  | **Labor monitoring** |  |  |
| FA93 | Was the woman in labor? | no=0, yes=1 | If no, go to  FA134 |
| FA94 | Was the partograph used? | no=0, yes=1 |  |
| FA95 | Stopping the dilation ? | Yes=1,  no = 0,  not documented= 9 | If no, go to FA97 |
| FA96 | If yes, duration of dilation stopping. | < 4hours = 1  From 4 to 6 hours= 2  > 6hours = 3  not documented = 9 |  |
| FA97 | Default of engagement at full dilation > 3 hours? | Yes=1,  no = 0,  not documented= 9 |  |
| FA98 | Induction of labor? | Yes=1,  no = 0,  not documented= 9 | If no, go to FA104 |
| FA99 | If yes, induction date | not documented=09 09 9999  (dd/mm/yyyy) |  |
| FA100 | If yes, induction time | not documented= 99 99  (hh:mm) |  |
| FA101 | Indication of tripping: multiple answers   - Maternal pathology - Fetal pathology - Post-term - Patient request - Other | (series of click)  *|__|*  *|__|*  *|__|*  *|__|*  *|__|* | If other, go to FA102 If no, go to FA103 |
| FA102 | If other specify | (open field) |  |
| FA103 | Failure of induction ? | Yes=1,  no = 0,  not documented= 9 |  |
| FA 104 | Active phase achieved? | no=0, yes=1 | If no go toFA108 |
| FA105 | Date start of active phase of labor | not documented=09 09 9999  (dd/mm/yyyy) |  |
| FA106 | Time start of active phase of labor | not documented= 99 99  (hh:mm) |  |
| FA107 | Duration of active phase of labor (hours) | not documented= 99  (numeric)  |__|__|hours |  |
| FA108 | Labour latency phase duration (in hours) | not documented= 99  (numeric)  |__|__|hours |  |
| FA109 | Signs of pre-rupture or uterine rupture? | Yes=1,  no = 0,  not documented= 9 |  |
| FA110 | A direction of laor has been attempted? | Yes=1,  no = 0,  not documented= 9 | If no, go to  FA120 |
| FA111 | Artificial rupture of membranes? | Yes=1,  no = 0,  not documented= 9  non applicable=8 (if FA78=1) | If no, go to FA120 |
| FA112 | If yes, date of artificial rupture of membranes | not documented=09 09 9999  (dd/mm/yyyy) |  |
| FA113 | If yes, time of artificial rupture of membranes | not documented= 99 99  (hh:mm) |  |
| FA114 | If yes, status of the amniotic fluid | Tinted = 1  Clear = 0  Not documented= 9 |  |
| FA115 | Delay realization caesarean after artificial rupture of membranes (RAM) | <4hours after ARM = 1  From 4 to 6 hours after ARM = 2  >6hours after ARM =3 | If answer = 1 go to FA120 |
| FA116 | Cervical dilation 4 hours after rupture of the membranes (in cm)? | not documented= 99  (numeric)  |__|__|cm |  |
| FA117 | Adequate uterine contractions (rhythm and intensity) 4 hours after rupture of membranes? | Yes=1,  no = 0,  not documented= 9 | If FA115=2 go to FA120 |
| FA118 | Cervical dilatation six hours after the rupture of membranes (in cm)? | not documented= 99,  (numeric)  |__|__| cm |  |
| FA119 | Were uterine contractions adequate (rhythm and intensity) 6 hours after rupture of membranes? | Yes=1,  no = 0,  not documented= 9 |  |
| FA120 | Anomaly of fetal heart rate during labor? | Yes=1,  no = 0,  not documented= 9 | If no go toFA126 |
| FA121 | If yes, start date of this anomaly | not documented=09 09 9999  (dd/mm/yyyy) |  |
| FA122 | If yes, start time of this anomaly | not documented= 99 99  (hh:mm) |  |
| FA123 | If yes, what was the treatment received? | Oxygenotherapy = 1  Lateral decubitus position = 2  Other measures = 6  No action = 3  Not documented = 9 | If other, go to FA124, If no go to FA125 |
| FA124 | If other treatment specify | (open field) |  |
| FA125 | Persistent anomaly after treatment? | Yes=1,  no = 0,  not documented= 9 |  |
| FA126 | Oxytocin? | Yes=1,  no = 0,  not documented= 9 | If no, go to  FA130 |
| FA127 | If yes, start date | not documented=09 09 9999  (dd/mm/yyyy) |  |
| FA128 | If yes, start time | not documented 99 99  (hh:mm) |  |
| FA129 | If yes, total dose infused before caesarean (in IU) | not documented 99 99  (numeric)  |__|__| IU |  |
| FA130 | Attempted instrumental delivery? | Yes=1,  no = 0,  not documented= 9 | If no, go to  FA134 |
| FA131 | If yes date of instrumental delivery attempt | not documented=09 09 9999  (dd/mm/yyyy) |  |
| FA132 | If yes attempt to instrumental delivery time | not documented 99 99  (hh:mm) |  |
| FA133 | If yes, technique employed | Forceps = 1  Vacuum = 2 |  |
|  | **Indication of caesarean section** |  |  |
| FA134 | Date indication caesarean | not documented=09 09 9999  (dd/mm/yyyy) |  |
| FA135 | Time indication caesarean | not documented 99 99  (hh:mm) |  |
| FA136 | Caesarean before labor? | Yes=1,  no = 0,  not documented= 9 |  |
|  | **Indication(s) of caesarean section** |  |  |
| FA137 | Fetal distress | no=0, yes=1 |  |
| FA138 | Long labor | no=0, yes=1 |  |
| FA139 | Preeclampsia | no=0, yes=1 |  |
| FA140 | History of caesarean | no=0, yes=1 |  |
| FA141 | If other indications, specify: multiple answers possible  - Placenta previa  - Placental abruption  - Abnormal or vicious presentation (front / transverse / hand / shoulder / posterior chin)  - Intrauterine growth restriction  - Post-term / term exceeded / prolonged pregnancy  - Vaginal bleeding late in pregnancy  - Multiple pregnancy / twin second retention  - Uterine Rupture  - Failed induction / caused dystocia  - Caesarean section for tubal sterilization  - Maternal request  - HIV  - Genital herpes / vulvar condylomas  - A history of rectal/ bladder / vaginal fistula  - pelvis suspect / pathological / unbalanced / limit / immature / narrow  - Precious pregnancy  - Prolapsed cord / funicular dystocia | (series of click)  *|__|*  *|__|*  *|__|*  *|__|*  *|__|*  *|__|*  *|__|*  *|__|*  *|__|*  *|__|*  *|__|*  *|__|*  *|__|*  *|__|*  *|__|*  *|__|*  *|__|* |  |
| FA142 | Signs of acute fetal distress at the moment of the indication of caesarean section? | Yes=1,  no = 0,  not documented= 9 |  |
| FA143 | Patient informed of the decision of caesarean? | Yes=1,  no = 0,  not documented= 9 |  |
| FA144 | Date incision | not documented=09 09 9999  (dd/mm/yyyy) |  |
| FA145 | Time incision | not documented 99 99  (hh:mm) |  |
| FA146 | Date extraction | not documented 09 09 9999  (dd/mm/yyyy) |  |
| FA147 | Time extraction | not documented 99 99  (hh:mm) |  |
| FA148 | Status newborn at birth or (T1) | Living =1  apparent death = 2  recent stillborn 3 =  macerated stillborn = 4  not documented = 9 |  |
| FA149 | Status T2 at birth | Living 1 =  apparent death = 2  recent stillborn 3 =  macerated stillborn = 4  not documented = 9  not applicable = 8 (if FA 61=no and FA 63=no) |  |
| FA150 | Status T3 at birth | Living =1  apparent death = 2  recent stillborn 3 =  macerated stillborn = 4  not documented = 9  not applicable = 8 (if FA63=no) |  |
| FA151 | Newborn or T1 resuscitate? | Yes=1,  no = 0,  not documented= 9 |  |
| FA152 | T2 resuscitate? | Yes=1,  no = 0,  not documented= 9  non applicable= 8 (if FA 61=no and FA 63=no) |  |
| FA153 | T3 resuscitate? | Yes=1,  no = 0,  not documented= 9  not applicable= 8(if FA63=no) |  |
| FA154 | If yes, duration of newborn or T1 resuscitation (in minutes) | not documented= 99  (numeric) |__|__| min |  |
| FA155 | If yes, duration of T2 resuscitation (in minutes) | not documented= 99  not applicable= 88 (if FA 61=no and FA 63=no)  (numeric) |__|__| min |  |
| FA156 | If yes, duration of T3 resuscitation (in minutes) | not documented= 99  not applicable= 88 (if FA63=no)  (numeric) |__|__| min |  |
| FA157 | Birth weight of newborn or T1 (in g) | not documented= 99 99  (numeric) |__|__|__|__| g |  |
| FA158 | Birth weight of T2 (en g) | not documented= 99 99  not applicable= 88 88 (if FA 61=no and FA 63=no)  (numeric) |__|__|__|__| g |  |
| FA159 | Birth weight of T3 (en g) | not documented= 99 99  not applicable= 88 88  (if FA63=no)  (numeric) |__|__|__|__| g |  |
| FA160 | Malformation ? | Yes=1,  no = 0,  not documented= 9 |  |
| FA161 | If yes, what type | (open field) |  |
|  | **Caesarean technique** |  |  |
| FA162 | Type of anesthesia | Spinal = 1 General = 2 Other = 6 |  |
| FA163 | If other, specify | (open field) |  |
| FA164 | Antibiotic prophylaxis? | Yes=1,  no = 0,  not documented= 9 | If no, go to  FA168 |
| FA165 | If yes, date of administration | not documented 09 09 9999  (dd/mm/yyyy) |  |
| FA166 | If yes, time of administration | not documented 99 99  (hh:mm) |  |
| FA167 | If yes, specify antibiotic used | Ampicillin=1  Amoxicillin=2  Amoxicillin+ clavulanic acid =3  Ceftriaxon=4  Other=6 | If other, go to FA168 If no, go to FA169 |
| FA168 | If other, specify | (open field) |  |
| FA169 | If yes, specify dose (in g) | (numeric) |__|g |  |
| FA170 | Type of cutaneous incision | Joel Cohen = 1  Median = 2  other = 6 |  |
| FA171 | If other specify | (open field) |  |
| FA172 | Type of uterine incision | Segmental = 1  Corporeal or segmento-corporeal = 2  Other = 6 | If other, go to FA173 If no, go to FA174 |
| FA173 | If other specify | (open field) |  |
| FA174 | Placental delivery mode | By controlled traction = 1 Manual = 2  Not documented = 9 |  |
| FA175 | Technique of parietal closure | According to Starck (closed in two layers) = 1  Layer by layer = 2  Other = 6 | If other, go to FA176 If no, go to FA177 |
| FA176 | If another technique, specify | (open field) |  |
|  | **Monitoring after caesarean** |  |  |
| FA177 | Regular blood pressure measurement two hours after caesarean section? | Yes=1,  no = 0,  not documented= 9 |  |
| FA178 | Diuresis measured after caesarean section? | Yes=1,  no = 0,  not documented= 9 |  |
| FA179 | Bleeding monitoring? | Yes=1,  no = 0,  not documented= 9 |  |
| FA180 | Patient eligible for anti thromboembolic prophylaxis? | Yes=1,  no = 0,  not documented= 9 | If no, go to  FA186 |
| FA181 | Anti thromboembolic prophylaxis conducted? | Yes=1,  no = 0,  not documented= 9 | If no, go to  FA186 |
| FA182 | If yes, start date of anti thromboembolic prophylaxis | not documented 09 09 9999  (dd/mm/yyyy) |  |
| FA183 | If yes, start time of anti thromboembolic prophylaxis | not documented 99 99  (hh:mm) |  |
| FA184 | If yes, what was the molecule used | Enoxaparin sodium = 1  Fraxiparin = 2  Calciparin = 3  Others = 6 |  |
| FA185 | If other specify molecule used | (open field) |  |
| FA186 | Liquid diet six hours after caesarean? | Yes =1,  no = 0,  not documented= 9 |  |
| FA187 | Discharge / death / transfer date | not documented 09 09 9999  (dd/mm/yyyy) |  |
| FA188 | Discharge / death / transfer time | not documented 99 99  (hh:mm) |  |
| FA189 | Status of the patient at discharge | alive =1  dead=0 |  |
| FA190 | Newborn or T1 status at discharge | alive = 1  dead before 24 hours = 2  dead after 24 hours = 3 |  |
| FA191 | T2 status at discharge | alive = 1  dead before 24 hours = 2  dead after 24 hours = 3  Not applicable = 8 (if FA 61=no and FA 63=no) |  |
| FA192 | T3 status at discharge | alive = 1  dead before 24 hours = 2  dead after 24 hours = 3  Not applicable = 8 (if FA63=no) |  |
